# Mini-encyclopedia of mitochondria-relevant nutraceuticals protecting health in primary and secondary care—clinically relevant 3PM innovation

**DOI:** 10.1007/s13167-024-00358-4

**Published:** 2024-04-18

**Authors:** Olga Golubnitschaja, Andrea Kapinova, Nafiseh Sargheini, Bianka Bojkova, Marko Kapalla, Luisa Heinrich, Eleni Gkika, Peter Kubatka

**Affiliations:** 1 Predictive, Preventive and Personalised (3P) Medicine, Department of Radiation Oncology, University Hospital Bonn, Rheinische Friedrich-Wilhelms-Universität Bonn, 53127 Bonn, Germany; 2 Biomedical Centre Martin, Jessenius Faculty of Medicine, Comenius University in Bratislava, 036 01 Martin, Slovakia; 3 Max Planck Institute for Plant Breeding Research, Carl-Von-Linne-Weg 10, 50829 Cologne, Germany; 4 Department of Animal Physiology, Institute of Biology and Ecology, Faculty of Science, P. J. Šafárik University in Košice, 040 01 Košice, Slovakia; 5 Negentropic Systems, Ružomberok, Slovakia; 6 PPPM Centre, s.r.o., Ruzomberok, Slovakia; 7 Institute of General Medicine, University of Leipzig, Leipzig, Germany; 8 Department of Radiation Oncology, University Hospital Bonn, Rheinische Friedrich-Wilhelms-Universität Bonn, 53127 Bonn, Germany; 9 Department of Histology and Embryology, Jessenius Faculty of Medicine, Comenius University in Bratislava, Martin, Slovakia

**Keywords:** Predictive preventive personalized medicine (PPPM / 3PM), Mitochondrial health, Mitophagy, Phytomedicine, Nutrients, Health protection, Biological age, Primary and Secondary care, Life quality, Longevity, Mitochondria-targeted substances, Sirtuins, Catechins, Vitamin B, Carnitine, L-Carnosine, Creatine, CoQ10, Resveratrol, Quercetin, Octacosanol, Pterostilbene, Alpha-lipoic acid, Senotherapeutics, Senolytics, Senomorphics, Fisetin, Piperlongumine, Curcumin, Kaempferol, Apigenin, Vitamin D, Silibinin, Allicin, Oleanolic acid, Ginseng, Melatonin, DMG-gold, Trehalose, *Ginkgo biloba*, Green tea, *Aloe vera*, Saffron, PQQ

## Abstract

Despite their subordination in humans, to a great extent, mitochondria maintain their independent status but tightly cooperate with the “host” on protecting the joint life quality and minimizing health risks. Under oxidative stress conditions, healthy mitochondria promptly increase mitophagy level to remove damaged “fellows” rejuvenating the mitochondrial population and sending fragments of mtDNA as SOS signals to all systems in the human body. As long as metabolic pathways are under systemic control and well-concerted together, adaptive mechanisms become triggered increasing systemic protection, activating antioxidant defense and repair machinery. Contextually, all attributes of mitochondrial patho-/physiology are instrumental for predictive medical approach and cost-effective treatments tailored to individualized patient profiles in primary (to protect vulnerable individuals again the health-to-disease transition) and secondary (to protect affected individuals again disease progression) care. Nutraceuticals are naturally occurring bioactive compounds demonstrating health-promoting, illness-preventing, and other health-related benefits. Keeping in mind health-promoting properties of nutraceuticals along with their great therapeutic potential and safety profile, there is a permanently growing demand on the application of mitochondria-relevant nutraceuticals. Application of nutraceuticals is beneficial only if meeting needs at individual level. Therefore, health risk assessment and creation of individualized patient profiles are of pivotal importance followed by adapted nutraceutical sets meeting individual needs. Based on the scientific evidence available for mitochondria-relevant nutraceuticals, this article presents examples of frequent medical conditions, which require protective measures targeted on mitochondria as a holistic approach following advanced concepts of predictive, preventive, and personalized medicine (PPPM/3PM) in primary and secondary care.

## Preamble

### Mitochondria as the life partner who insists in healthy metabolism—attributes for the paradigm change from reactive medical services to 3PM

In order to meet organismal needs and maintain physiological homeostasis, concerted metabolic pathways keep a control over physiologic bioavailability of essential biomolecules which have to be available at the right time and in appropriate concentration. The overall process coordinates the life-important balance between anabolism and catabolism, namely,

- On the one hand, biosynthetic pathways generating macromolecules by utilizing energy stored in a form of ATP (adenosine triphosphate) and NADPH (nicotinamide adenine dinucleotide phosphate) to generate macromolecules
- On the other hand, catabolic pathways breaking down macromolecules into the pool of elements to cover the demand on building materials for anabolism and energy production

Both parts of the whole depend directly on the healthy mitochondrial population as the powerhouse at cellular and organismal levels.

Despite their subordination in humans as a subcellular component of eucaryotic cells, to a great extent, mitochondria maintain their independent status over the entire history of symbiosis with humans, e.g., by keeping their genetic identity (mtDNA). Further, the high dynamicity of their homeostasis (fission versus fusion and mitophagy) allows mitochondrial population for promptly adapting under changing environment to remain within their comfort zone despite health risks at systemic level. Further, mitochondria and humans demonstrate life-important synergies: all compensatory mechanisms indicate that they learned to cooperate together on protecting life quality and minimizing health risks. Under oxidative stress conditions, healthy mitochondria promptly increase mitophagy level to remove damaged “fellows” rejuvenating the mitochondrial population and sending fragments of mtDNA as SOS signals to all systems in the human body. As long as metabolic pathways are under systemic control and well-concerted together, adaptive mechanisms become triggered increasing systemic protection, activating antioxidant defense and repair machinery [1]. Contextually, all attributes of mitochondrial patho-/physiology are instrumental for predictive medical approach and cost-effective treatments tailored to individualized patient profiles in primary (to protect vulnerable individuals again the health-to-disease transition) and secondary (to protect affected individuals against disease progression) care [2].

The cause of compromized mitochondrial health ranges from preventable risks such as suboptimal lifestyle and dietary patterns (majority of affected individuals) to inherited or acquired mutations in chrDNA or mtDNA (mitochondrial diseases which create a minor portion). All systems in human body suffer from compromized mitochondrial health but the most affected are highly energy-consuming ones which are the heart, the central and peripheral nervous system, and skeletal and smooth muscles resulting in frequently fatal cardiac deficits (heart failure, sudden heart arrest), accelerated brain aging/neurodegeneration, and a spectrum of muscular pathologies. Further, also the kidney and pancreatic β-cells producing insulin belong to the tissues which are highly susceptible to defective mitochondrial oxidative phosphorylation [3]. Impairments specifically in the mitochondrial oxidative phosphorylation can be caused by low energy and oxygen supply, e.g., under ischemic conditions and deficits of electron transport chain (ETC) enzymes and of electron carrier CoQ_10_. To this end, corresponding toxic effects are abundantly described, for example, for the alcohol abuse which inhibits mtDNA replication. Further, cocaine, cyanide, chloroform, acid, cholic acids, among others, are known to inhibit ETC enzymatic complexes I–IV. Above a certain threshold, the damage is irreversible, and the lack of ATP and uncoupled mitochondria are fatal for the affected cells then triggering apoptosis [3]. However, below the threshold, targeted application of mitochondria-related nutraceuticals may reverse the damage and stabilize corresponding conditions. For example, *Ginkgo biloba* stimulates ETC enzymatic complexes I and III, whereas melatonin stimulates ETC enzymatic complexes I and IV. Further, dietary CoQ_10_ supplementation is recommended to treat cardiomyopathy-associated deficits as well as to compensate its plasma level concentrations usually reduced by therapeutical application of statins.

Mitochondrial diseases are highly heterogeneous from genetic and clinical points of view with no curation possible. Therapeutic strategies utilize a number of nutraceuticals to milden disease severity in corresponding medical conditions. To this end, CoQ_10_ and vitamins B_1_ and B_2_ are recommended to support ETC efficacy, whereas creatine is used for energy buffering. Further, epicatechin is considered effective to stimulate mitochondrial biogenesis. Arginine and citrulline are used to restore nitric oxide production. Cysteine donors, vitamins C and E, and lipoic acid are utilized due to their antioxidant properties. Overall, individualized application of nutraceuticals is considered a promising approach to mitigate mitochondrial impairments [4].

### Demand on individualized application of nutraceuticals in primary and secondary care

Nutraceuticals, a hybrid of nutrition and pharmaceuticals, also known as phytochemicals and functional foods, are naturally occurring bioactive chemical compounds demonstrating evidence-based health-promoting, illness-preventing, and other health-related benefits. Health-promoting properties of nutraceuticals along with their great therapeutic potential and safety profile have made them to the robust source of novel drugs and food industry products, as well as herbal and dietary supplements in traditional and innovative medical approaches [5]. Keeping in mind these huge advantages, there is a permanently growing demand on individually adapted application of nutraceuticals in primary and secondary care.

### Multi-faceted beneficial effects of flavonoids are linked to mitochondrial health and bioactivities affecting all functions at cellular and organismal levels

Flavonoids are naturally synthesized as bioactive secondary metabolites in various plant foods such as fruits, vegetables, nuts, whole grains, and medicinal herbs [6]. More than 10,000 different flavonoid compounds have been isolated and characterized regarding their health-promoting effects; their beneficial effects are evidence-based and well-documented [7]. Depending on their chemical structure, flavonoids are divided into seven subfamilies: flavones, flavanones, flavonols, flavan-3-ols, isoflavones, anthocyanidins, and chalcones. The majority of these compounds possess strong antioxidant and radical scavenging, vasodilating/antihypertensive, anti-inflammatory, immunomodulatory, antiangiogenic, anticancer, antiviral, antigenotoxic, antiallergenic, and antidepressant-like properties—all are of great clinical relevance [8, 9]. Their health beneficial effects are applicable early in life, e.g., in pregnant women diagnosed with preeclampsia [6]: targeted application of flavonoids suppresses pro-inflammatory pathways positively affecting vascular system by antihypertensive, antiatherogenic, antioxidant, antiplatelet, and vasodilating actions, thereby mitigating risks of maternal and fetal mortality and improving long-term individual outcomes [10]. Directly or indirectly, beneficial health effects of flavonoids are linked to physiologic mitochondrial homeostasis and/or bioactivities affecting all functions at cellular and organismal levels [11–13]. Since flavonoids have poor oral bioavailability, the gut microbiome plays a significant role in their absorption and metabolism. It is estimated that only 5–10% of dietary flavonoids are absorbed in the small intestine. The remaining 90% of consumed flavonoids are generally degraded via enzymatic processes by the large intestine’s resident microbiota or excreted from the body; therefore, technological solutions which would allow for increased bioavailability of flavonoids are extensively under consideration by multi-professional groups involved [10].

## Mitochondrial sirtuins

Sirtuin is a protein family controlling senescence and longevity relevant processes by epigenetic regulation and balancing together the anabolism (biosynthesis process utilizing energy from ATP and NADPH to generate macromolecules) versus catabolism (the breakdown of macromolecules into smaller compounds further used to fuel anabolism). Therefore, biological age to a large extent depends on the sirtuin functionality. The sirtuin family includes seven members SIRT1–SIRT7, of which SIRT3, SIRT4, and SIRT5 are predominantly localized at mitochondria and therefore referred to as mitochondrial sirtuins which bridge together metabolism, senescence, and longevity [14].

The silent information regulator 2 (Sir2) gene was identified in *Saccharomyces cerevisiae* for the first time. A study showed that it prolonged budding yeast’s life span by inhibiting genome instability [15]. *SIR2*-like genes, known as sirtuins, are abundant in most organisms, such as plants, bacteria, and animals, and contribute to their health and survival [16]. The demonstration that sirtuins function as NAD + -dependent protein deacetylases (deacylases) opened up a novel avenue of research into the metabolic modulation of sirtuins and regulation of their activity by small molecules [15]. As a result of deacetylation, cellular NAD + metabolism is integrated into a wide array of cellular mechanisms like cell metabolism, cell survival, cell cycle, cell death, DNA repair, mitochondrial oxidative metabolism and the consequent resistance to oxidative stress, and even life expectancy. The most widely investigated sirtuin, SIRT1, is located in the nucleus and cytosol and, along with histone deacetylation, controls transcription factors, such as p53, NF-κB, forkhead boxes (FOXOs), peroxisome proliferator-activated receptor γ coactivator 1-α (PGC1α), and DNA repair proteins, such as PARP1. SIRT2 is a cytosolic sirtuin, while SIRT3, 4, and 5 are present in mitochondria and involved in oxidative stress and lipid metabolism. SIRT6 and 7 are in the nuclei and play a role in gene expression and DNA repair. Age-related reductions in NAD + levels and sirtuin activity are contributing factors in the pathogenesis of a wide variety of cardiovascular and metabolic disorders, including atherosclerosis, endothelial dysfunction, acute cardiac syndromes, cardiomyopathy, hypertrophy and heart failure, arrhythmias, hypertension, metabolic syndrome, obesity, fatty liver, diabetes mellitus, and dyslipidemia [17]. A study in mice demonstrated that ageing is associated with an increase in CD38 activity, which is negatively correlated with NAD + levels and mitochondrial activity. In part, this response can be explained by a decline in SIRT3 activity. In light of the decline of sirtuin activity, the loss of NAD + levels is the primary cause of sirtuin inactivity, senescence, and ageing-related diseases [18].

## PPAR-PGC-1 alpha axis

The PPAR-PGC-1α axis is a potential therapeutic target for the regulation of mitochondrial biogenesis and function in multiple serious diseases.

The peroxisome proliferator-activated receptor gamma coactivator 1-alpha (PGC-1α) is a protein encoded by the *PPARGC1A* gene with a central role in the regulation of mitochondrial biogenesis and function. It is highly expressed in mitochondria-enriched tissues with high energy demands. Disorders in its expression, function, or PGC-1α-related pathways are significantly associated with the metabolic syndrome, pathogenesis of obesity, type 2 diabetes mellitus, cardiovascular and liver diseases, or cancer [19–21]. The peroxisome proliferator-activated receptor alpha (PPARα) is a ligand-activated transcription factor of the pleiotropic nuclear receptor 1C (NR1C) family (also known as the PPAR family) [22, 23]. In addition, they are known further isoforms of PPAR receptors, i.e., the peroxisome proliferator-activated receptors gamma (PPARγ) and beta/delta (PPARβ/δ). Together, they are significantly involved in the metabolism of lipids and glucose in various tissues, and their dysregulation is associated with several diseases, including cardiovascular diseases, Alzheimer’s disease, or diabetes mellitus. PPARα is particularly highly expressed in hepatocytes, cardiomyocytes, proximal renal tubular cells, and brown adipocytes, whereas PPARβ/δ in the cells of skeletal muscle, skin, adipose tissue, heart, or liver [23].

The PPARα-PGC-1α axis plays an important role in the regulation of cardiac function. In the myocardium, it controls fatty acid and glucose metabolism and mitochondrial biogenesis as well as mitochondrial energy metabolism [24, 25]. Research has shown that the expression levels of PPARα and PGC-1α were reduced in various experimental models of heart failure [25, 26]. The PPARβ can promote mitochondrial biogenesis by increasing expression of the nuclear respiratory factor 1 (NRF-1) and by stabilization of existing PGC-1α protein [27, 28]. It turned out that the PGC-1α/PPARβ axis has also an essential role in the induction of the uncoupling protein 3 (UCP3) expression, one of the important mitochondrial membrane proton transporters (UCPs) regulating lipid metabolism and mitochondrial production of reactive oxygen species [29]. In the study of Lima et al. [29], the UCP3 protein expression affected fatty acid metabolism, PGC-1α-induced oxidative capacity, and the adaptive mitochondrial response to fatty acid exposure in skeletal muscle cells. In addition, the PPAR-PGC-1α axis is also a significant regulator of mitochondrial biogenesis in many other vital organs, including the brain, liver, kidney, or adipose tissue [25, 28, 30].

Beneficial pharmacological activity of synthetic PGC-1α or PPARα agonists (such as, e.g., fibrates and thiazolidinediones) has been shown, but their adverse effects in various systems are also known [25, 26, 31]. Conversely, numerous preclinical studies reported the beneficial effects and safety of using PGC-1α or PPARα agonists occurring in natural sources, such as polyphenols, terpenoids, and polyacetylenes [30–32]. Quercetin demonstrated cardio- and neuroprotective effects by regulating the PPAR and PGC-1α axis in several studies [33–35]. Epigallocatechin gallate (EGCG) upregulated PGC-1α and decreased oxidative stress in in vitro Parkinson’s disease model [36]. In other experimental studies, hepatoprotective effects of several natural compounds (resveratrol, rosmarinic acid, astaxanthin, neohesperidin) were shown in this context [37–40].

## Senotherapeutics

Among mitochondria-targeting substances, senotherapeutic drugs have drawn a great deal of attention in a search of strategies to extend the healthspan and to prevent or treat age-related pathologies. Senotherapeutics include three classes: senolytics which selectively eliminate senescent cells; senomorphics, which modulate or reverse senescent cell phenotype; and mediators of the immune system clearance of senescent cells [41].

The first report on senolytics comes from 2015. Zhu et al*.* [42] found that quercetin (a natural product) and dasatinib (a synthetic product) eliminated senescent mouse embryonic fibroblasts and extended healthspan in mice. Since then, numerous reports on modulating senescence using senotherapeutics were published. Many of them belong to polyphenol group and have attracted attention mostly due to involvement in molecular pathways crucial for maintaining viability of tumor cells. Preclinical evidence shows that polyphenols exert greater selectivity in tumor cells to target oxidative phosphorylation and regulation of the mitochondrial membrane potential (MMP), glycolysis, pro-oxidant pathways, and antioxidant (adaptive) stress responses and their anticancer efficacy relates to the protonophoric and pro-oxidant properties rather than the specific effects on downstream molecular targets affected by MMP dissipation and mitochondrial uncoupling [43].

Below, naturally occurring senolytics (quercetin, fisetin, piperlongumine, and curcumin) and senomorphics (resveratrol, kaempferol, and apigenin) are considered followed by other bioactive substances in the context of senescence and antiageing.

### Quercetin

Quercetin (2-(3,4-dihydroxyphenyl)-3,5,7-trihydroxy-4H-chromen-4-one), a flavonoid from a flavonol group, and its derivatives possess antioxidant properties and strongly influence cellular lifespan, survival, and viability. A series of preclinical studies demonstrated antiageing and rejuvenating effects of quercetin [44]. Specifically in senescent fibroblasts, improved homeostasis and significant rejuvenation have been reached by quercetin supplementation. The mechanisms of the observed rejuvenation rely on the Sirt1-agonist function of quercetin and restoring physiologic levels of mitophagy and renewing functional populations of mitochondria [1]. Strong health beneficial effects of quercetin supplementation are demonstrated in the reproductive medicine–related research such as overall quality, viability, and motility of sperm linked to improved parameters of the mitochondrial health [45].

Quercetin is a flavonoid demonstrating strong senolytic effects and promoting mitophagy. Glycosidic forms of quercetin are found in a variety of plants, particularly in fruits and vegetables. Major sources are capers (3 mg/g) [46] and onions (0.3 mg/g) [47]; others include chili peppers, apples, apricots, grapes, berries and tea, and some medicinal plants (*e.g.*, *Euphorbia helioscopia*) [48–50].

Numerous beneficial effects of quercetin were reported in vitro and in vivo—anti-inflammatory, anticancer, antidiabetic, cardioprotective, and neuroprotective; most of them are attributed to its antioxidant activity. Antifungal, antibacterial, and antiviral activities of quercetin were reported as well [51, 52]. Quercetin maintains oxidative homeostasis by regulating the non-enzyme-dependent and enzyme-mediated antioxidant defense system and also through targeting signaling pathways induced by ROS (reactive oxygen species) [53]. These pathways include PI3K/Akt/mTOR (phosphatidylinositol 3-kinase/protein kinase B/mammalian target of rapamycin), AMPK/SIRT1/NF-κB (AMP-activated protein kinase/NAD-dependent deacetylase sirtuin-1/nuclear factor kappa B), and MAPK/AP-1 (mitogen-activated protein kinase/activator protein 1), which play role in many human pathologies like cancer, diabetes, neurodegenerative, and cardiovascular disorders [54–57].

Involvement of quercetin in mitochondrial processes is well-documented [58]. Preclinical research showed that quercetin supports mitophagy and modulates mitochondrial biogenesis, MMP, oxidative respiration, and ATP anabolism as well as intramitochondrial redox status [50]. Quercetin has also drawn attention due to purported improvement of endurance exercise capacity. Quercetin upregulates SIRT1 and PGC-1α, increases mtDNA and cytochrome c content in the muscle and brain, increases endurance performance in rodents [59], and prevents muscle atrophy in denervated mice [60], although, for example, an exercise performance is not influenced by quercetin supplementation in humans [61]. Quercetin combined with dasatinib synergistically decreases senescent cell burden in patients with diabetic kidney disease [62].

In summary, data collected regarding health benefits of quercetin are encouraging. Currently, a clinical study evaluating the effect of quercetin and another senolytic substance fisetin on skeletal health in older subjects is ongoing [63]. It must be considered, though, that in general population, the direct absorption of the quercetin glycosides from plant sources is negligible and the hydrolysis to more lipophilic aglycones is necessary for absorption. Further, the bioavailability of senolytics depends on the type of glycoside [50], individual genetic polymorphisms, composition of gut microbiota, and drug exposure, among others [64]. Improved quercetin formulations for oral administration, *e.g.*, based on lecithin with better solubility and absorption, have been proposed [65].

### Fisetin

Fisetin (5-deoxyquercetin; 3,3′,4′,7-tetrahydroxyflavone; 2-(3,4-dihydroxyphenyl)-3,7-dihydroxy-4H-chromen-4-one) is another flavonol commonly found in fruits and vegetables, particularly in strawberries (160 µg/g) and apples (27 µg/g), mostly in glycoside form; other significant sources include persimmon, lotus root, onions, grapes, and kiwi [47, 66]. Fisetin acts as an antioxidant through multiple mechanisms, by scavenging ROS/RNS (reactive nitrogen species), restraining oxidant enzymes, alleviation of oxidative stress induced by nitric oxide, reinforcing enzymatic and non-enzymatic intracellular antioxidants, chelating with transitional metal ions, acting as a substrate for oxidoreductase activity, and interaction with redox-related signaling pathways [67]. Antioxidant properties are implicated in its anti-inflammatory activity [68] and cardioprotective [69] and neuroprotective [70] effects. Further, its antidiabetic properties were reported: fisetin improves glucose homeostasis by direct inhibition of liver gluconeogenesis [71].

It seems that modulation of cell signaling pathways by fisetin differs in cancer cells compared to normal cells [72]. Fisetin protects cells from apoptosis by targeting both intrinsic and extrinsic apoptotic pathways and enhances proliferation through upregulation of cell cycle activators [69]. In cancer cells, however, fisetin exerts antiproliferative and pro-apoptotic effects via activation of both death receptor and mitochondrial-dependent pathways [73, 74] and inhibits cell migration, metastatic activity, and angiogenesis [75–77]. The anticancer properties of fisetin can be attributed to regulation of several signaling pathways, including MAPK, NF-κB, PI3K/Akt/mTOR, vascular endothelial growth factor, and Nrf2/HO-1 (transcription factor nuclear factor erythroid 2-related factor 2/heme oxygenase 1) [78].

Fisetin treatment upregulates PGC-1α and TFAM (mitochondrial transcription factor A) mRNA levels, increased mtDNA copy number, and mitochondrial mass in neuronal cells. Fisetin also modulates expressions of anti-Parkinson’s molecular panels involved in neuroprotection and neuronal differentiation [79]. In vivo fisetin increases mitochondrial ATP production in ischemia–reperfusion insult hearts by activating electron transport chain complex 1, thus preserving the function of interfibrillar mitochondria [80]. Fisetin improves mitochondrial dysfunction and impairment in palmitate-treated hepatocytes and attenuated non-alcoholic fatty liver disease in mice fed a high-fat diet [81]. A rigorous study from 2018 [82] showed that out of the 10 flavonoids tested (including resveratrol, quercetin, ECGC, and curcumin), fisetin had the most potent senolytic effects against several cell types in vitro. Fisetin reduced markers of senescence and senescence-associated secretory phenotype in multiple tissues, extended the healthspan, and prolonged the lifespan in mice [82].

Preclinical research revealed the beneficial effects of this flavonol in neurodegenerative, cardiovascular, metabolic, and cancer diseases, but these results have to be verified in human subjects. A clinical trial showed that fisetin may improve the treatment of brain ischemic stroke [83]. Other clinical trials evaluating the effects of fisetin on vascular function in older adults and alleviation of frailty in older subjects or in cancer survivors are currently underway [84–87]. It has to be considered, though, that the bioavailability of fisetin after oral administration is poor, so it is necessary to develop an effective delivery method to enhance its therapeutic potential in treatment of a particular disease, *e.g.*, using nanotechnologies [88].

### Piperlongumine (piplartine)

Another senolytic substance is piperlongumine (or piplartine) (1-[(2E)-3-(3, 4, 5-trimethoxyphenyl) prop-2-enoyl]-1, 2, 5,6-tetrahydropyridin-2-one), a natural amide alkaloid found in *Piper* species. A *cis* isomer and *trans* isomer were identified; the latter is more stable and showed higher cytotoxic activity in cancer cells in a dose-dependent manner, whereas the *cis* isomer was inefficient [89].

Piperlongumine has drawn attention mostly to its anticancer activity attributed to modulation of various pathways, *e.g.*, PI3K/PKB/mTOR, NF-κB, JNK/STAT3 (Janus kinases/signal transducer and activator of transcription 3). Piperlongumine affected various hallmarks of cancer such as cell survival, proliferation, invasion, angiogenesis, epithelial-mesenchymal-transition, and metastases and also inhibited radioresistance and chemoresistance of cancer cells [90–92]. Piperlongumine targeted various types of cancer cells selectively, with minimal toxicity to normal cells [93, 94]. Piperlongumine showed cytotoxic effects in human cancer cells via increased intracellular ROS production and subsequent mitochondrial dysfunction [90, 95–97]. Piperlongumine reduced the migration and colony formation in breast cancer cells through multiple mechanisms including modulation of glucose import, NF-κB activation, and lncRNA expression. In the same study, piperlongumine also enhanced the efficacy of doxorubicin on mammary tumor growth in vivo [92]. Piperlongumine inhibited experimental colon cancer via Ras/PI3K/Akt/mTOR signaling axis and induced mitochondrial apoptotic pathway by downregulating Bcl-2 levels [93]. Inhibition of cell growth and induction of apoptosis by piperlongumine were also reported in human melanoma cells, via ROS-mediated mitochondria disruption and JNK (p-Jun N-terminal kinase) pathway [90]. Apart from anticancer effects, antinociceptive, anxiolytic, antidepressant, antiatherosclerotic, antidiabetic, antibacterial, antifungal, leishmanicidal, trypanocidal, and schistosomicidal activities were reported too [98].

Human data on piperlongumine are missing, apart from a single clinical study in Japanese women which showed that *Piper longum* extract increased peripheral blood flow and skin temperature after cold stress [99]. Piperlongumine seems to be very safe in vivo [98]; however, the limited bioavailability and hydrophobicity restrict its application. More efficient delivery methods, *e.g.*, using nanoformulation, may result in better therapeutic effect [100]. A drug interaction has to be considered with piperlongumine administration as it was reported to inhibit CYP1A2 isoenzymes [101].

### Curcumin

Curcumin, a (1E,6E)-1,7-bis(4-hydroxy-3-methoxyphenyl)-1,6-heptadiene-3,5-dione, is present in the rhizome of *Curcuma longa*. It is one of the primary components in turmeric and curry ingredients that are often used as flavoring agents in Asian and Middle Eastern countries [102]. Mango ginger, also known as *Curcuma amada*, belongs to the ginger family Zingiberaceae. It contains curcuminoids and curcumin but in much smaller amounts compared to turmeric [103].

Curcumin shows significant beneficial effects on human health, which include anti-inflammatory and antioxidative effects. Moreover, curcumin has substantial neuroprotective activities against a broad spectrum of xenobiotics [104]. In addition, curcumin has been proven to control hyperlipidemia, metabolic syndrome, arthritis, and anxiety [105]. Cumulative research data pointed to curcumin as a molecule which elevates mitochondrial functions in various preclinical and clinical conditions [106, 107].

The neuroprotective effects of curcumin via effects on mitochondrial functions are well-documented. Curcumin demonstrated protective activities against oxygen–glucose deprivation/reoxygenation-induced injury in astrocyte primary cells in vitro through the elevated mitochondrial function and triggering the ERK signaling pathway [108]. In another study, curcumin demonstrated neuroprotective activities against amyloid-β-induced mitochondrial dysfunction which were associated with the inhibition of the GSK-3β signaling pathway [109]. Miao et al. [110] described that curcumin administration suppressed inflammation and mitochondrial dysfunction in experimental stroke through the activation of Sirt1 cell signaling. Anti-inflammatory activity and positive effects on the mitochondrial function of curcumin were documented in the study using a liver steatosis model in obese mice [111]. Curcumin affected several mechanisms of action such as nuclear respiratory factor 1 (NRF1) and mitochondrial transcription factor A (TFAM) gene signaling and reduced hepatic NF-κB signaling. Besides that, curcumin normalized mtDNA and restored mitochondrial oxidative metabolism and biogenesis [111]. Regarding the metabolic activities of curcumin, it improved mitochondrial dysfunction which correlated with the suppression of gluconeogenesis in free fatty acid–regulated hepatic lipoapoptosis [112]. Curcumin also upregulated cell signals regulating mitochondrial biogenesis, i.e., including peroxisome proliferator-activated receptor-γ coactivator 1α (PGC1α), NRF1, and TFAM. In the model of osteoarthritis, curcumin was documented as an activator of mitophagy in preserving mitochondrial function (ROS, Ca^2+^, ATP synthesis, and mitochondrial membrane potential). In this study, curcumin showed significant chondroprotective effects in the model of osteoarthrosis via the activation of the AMPK/PINK1/Parkin cell signaling [113].

Most, if not all, cell types in the organism, including the cardiovascular and nervous system, depend on appropriate mitochondrial function. It has been described that curcumin has outstanding neuro- and mitochondria-protective effects against wide-spectrum neurotoxic substances involved in the multi-step process of neurodegenerative diseases’ development [104]. Curcumin has considerable clinical potential in other chronic diseases such as cardiovascular and metabolic diseases or cancer [114–116]. For the introduction of curcumin into clinical practice, more standardized clinical trials are required to fully uncover its preventive and therapeutic potential in clinical practice.

### Resveratrol

One of the most known polyphenols, resveratrol from the stilbene group (3,4′,5-trihydroxystilbene, 5-[(E)-2-(4-hydroxyphenyl) ethenyl] benzene-1,3-diol) is a phytoalexin produced by a variety of plants and herbs in stress response. Human dietary resveratrol sources include grapes (8 µg/g), wine (98–1800 µg/100 mL), berries, peanuts, soy, and also Japanese knotweed known as Itadori tea (2 mg/g in root) [117, 118]. Numerous molecular targets of resveratrol were reported, most important follow. Resveratrol activates AMPK [119, 120]; downregulates NF-κB [121, 122] thus impacting numerous cellular processes, particularly inflammatory responses, cellular growth, and apoptosis [123, 124]; and downregulates PI3K/Akt and mTOR pathway [125, 126], involved in regulating a variety of processes including insulin signaling, cell proliferation, and survival [127]. Resveratrol can modulate mitochondrial calcium, increase the efficacy of chemotherapeutic agents, target cancer stem cells, alleviate inflammation associated with tumors, and promote the elimination of impaired mitochondria through mitophagy [128, 129]. Resveratrol was also shown to suppress cytochrome P450 enzymes which activate procarcinogens to carcinogens [130, 131]. Apart from anticancer, antidiabetic, cardioprotective, and neuroprotective effects, antimicrobial activity of resveratrol was reported too [132].

Mitochondrial impairment–related disorders are ameliorated by resveratrol mainly through the redox-dependent mechanisms. Resveratrol upregulates mitochondrial antioxidative enzymes and triggers mitochondrial biosynthesis, thus acting as mitochondrial protective substance [133, 134]. Unfortunately, human data focusing on the impact of the resveratrol on mitochondrial function are scarce, with limited number of subjects involved. Resveratrol supplementation in obese patients mimicked the effects of caloric restriction—activation of AMPK, increase in SIRT1 and PGC-1α protein levels, increase in citrate synthase activity without change in mitochondrial content, and improved muscle mitochondrial respiration were found [135]. However, positive effect of resveratrol on muscle mitochondrial function was not confirmed by other reports [136, 137]. Resveratrol ameliorated mitochondrial respiratory dysfunction in patient-derived fibroblasts carrying homoplasmic mtDNA mutations [138]. Resveratrol triggered apoptosis via the involvement of mitochondrial pathway in fibroblast-like synoviocytes derived from patients with rheumatoid arthritis [139] and was proved as an effective adjuvants in the management of rheumatoid arthritis patients [140]. Improvement of skeletal muscle mitochondrial functions was seen in older patients when resveratrol was combined with exercise [141]. An ongoing clinical study investigates the effect of 12-week resveratrol administration on skeletal muscle mitochondrial function in people with type 1 diabetes [142].

Safety concerns regarding resveratrol supplementation emerged as some preclinical reports showed that, depending on the concentration and the cell type, resveratrol may also amplify oxidative stress [143] and induce mitochondrial dysfunction associated with bioenergetic impairments and/or apoptosis triggering [144]. So, despite large evidence of beneficial effects of resveratrol in diseases associated with mitochondrial dysfunction (*e.g.*, Alzheimer’s disease, Parkinson’s disease, muscle atrophy, cardiovascular disease, arthritis, obesity, and cancer) [129], adverse effects in normal cells have to be considered [145]. In addition, doses proved to be efficient in preclinical research, *e.g.*, against cancer cells by in vitro assays, largely exceed those which may be achieved by dietary intake, and, moreover, resveratrol possesses a low bioavailability as it is rapidly metabolized [146]. Resveratrol-drug interaction using high dosage due to inactivation of CYP450 isoenzymes is also of concern [147]. Therefore, the dose and formulation of resveratrol for human use should be carefully considered.

### Kaempferol

Kaempferol (3,4′,5,7-tetrahydroxyflavone, 3,5,7-trihydroxy-2-(4-hydroxyphenyl) chromen-4-one) is a natural flavonol occurring in a variety of plants and plant-derived foods; the highest content was found in capers (ca 1 mg/g); other good sources include cloves, cumin, dill, kale, broccoli, garden cress, and spinach and also black tea infusion (ca 1 mg/100 mL) [148].

Kaempferol displays strong antioxidant capacity through reduction of ROS [149–151]. Attenuation of oxidative stress relates to anti-inflammatory properties through modulation of the NF-κB pathway [152]. Vast evidence exists regarding its antitumor potential which includes regulation of cell cycle, apoptosis induction (via mitochondrial-dependent pathway), and inhibition of angiogenesis. Kaempferol induced mitochondrial dysfunction and mitophagy in breast precancerous lesions both in vitro and in vivo; reactivation of STK11 (serine/threonine kinase)/AMPK pathway was required for excessive mitochondrial fission and lethal mitophagy [153]. Other pathways were reported to be involved in anticancer activity of this flavonol, *e.g.*, ERK (extracellular signal-regulated kinase)/p38 MAPK and PI3K/Akt/mTOR STAT3, transcription factor AP-1, and Nrf2 [154, 155]. Kaempferol also inhibited melanoma metastasis via blocking aerobic glycolysis of melanoma cells [156]. In addition, kaempferol showed antidiabetic [157], cardioprotective [158], neuroprotective [159, 160], osteoprotective [161], antimicrobial [162], and antiviral properties too [163]. Kaempferol had a protective effect against a cisplatin-induced acute ovarian damage in mice [164] and protected cardiomyocytes against hypoxia/reoxygenation-induced injury via promoting Notch1/PTEN/Akt signaling pathway [159].

Kaempferol improved mitochondrial function in lung-ischemia reperfusion injury both in vitro and in vivo*.* Kaempferol increased the cell viability, improved MMP, inhibited the opening of mitochondrial permeability transition pores, reduced the levels of oxidative stress and apoptosis, increased the expressions of Bcl-2 and mitochondrial cytochrome c, and decreased the expressions of Bax and cytoplasmic cytochrome via SIRT 1/PGC-1α signaling pathway [165]. Upregulation of the mitochondrial quality control proteins PGC-1α and also PINK1, Parkin, and Beclin was reported in another study [151].

Preclinical evidence regarding beneficial health effects of kaempferol is promising but human data are scarce. Epidemiological studies showed that high intake of kaempferol is associated with non-significant decrease in incidence of several types of cancer; however, potential confounding factors could bias estimates of risk [166]. Clinical studies are needed to elucidate the association between kaempferol intake and incidence of cancer and other pathologies, but first, it is necessary to evaluate the safety of its excessive use. A recent randomized, placebo-controlled trial showed that the consumption of 50 mg kaempferol aglycone daily for 4 weeks is safe in healthy adults [167]. The bioactivity of kaempferol, which depends on the ingested conjugate, must be considered; however, human reports on the bioavailability of dietary kaempferol are missing [168].

### Apigenin

Apigenin (4′,5,7,-trihydroxyflavone, 5,7-dihydroxy-2-(4-hydroxyphenyl) chromen-4-one) is one of the most abundant flavonoids in plants from the flavone subclass. It is found in the species from the Apiaceae, Asteraceae, Lamiaceae, and the Fabaceae family; best dietary sources are parsley (ca 45 mg/g in the dried form), chamomile (3–5 mg/g in the dried form), celery, vine spinach, artichokes, and oregano*.* Apigenin occurs in the aglycone form and/or its glucosides, glucuronides, methyl ethers, and acetylated derivatives [169, 170].

Apigenin displays antioxidant activity through inhibition of pro-oxidant enzymes, modulation of redox signaling pathways (NF-κB, Nrf2, MAPK, and P13/Akt), reinforcing enzymatic and non-enzymatic antioxidants, metal chelation, and free radical scavenging [171]. Suppression of NF-κB activation and subsequent downregulation of NF-κB gene products involved in inflammation [172], proliferation (*e.g.*, cyclin D1, COX-2), apoptosis, and angiogenesis [173] elucidate some of the mechanisms of apigenin antitumor activity which was extensively reported [174, 175]. As other polyphenols, apigenin seems to selectively target mitochondria in cancer cells; in a rat model of hepatocellular carcinoma, apigenin increased MMP, ROS level, mitochondrial swelling, and cytochrome c release leading to apoptosis only in cancerous hepatocytes [176]. Other beneficial effects of apigenin include antidiabetic [177], cardioprotective [178], neuroprotective [179], antibacterial [180], and antiviral [181].

Vast evidence exists on apigenin alleviation of various pathologies by targeting mitochondria. He et al*.* [182] reported that apigenin induced mitochondria-dependent apoptosis in hypoxic pulmonary artery smooth muscle cells and protected against pulmonary hypertension via inhibition of the hypoxia-inducible factor 1α–KV1.5 channel pathway. Apigenin has a potential to alleviate non-alcoholic fatty liver disease; a decrease of hepatic lipid accumulation by activating the autophagy-mitochondrial pathway in human hepatoma cells was reported [183]. In an animal model of acute myocardial infarction, apigenin attenuated cardiomyocyte injury by modulating Parkin-mediated mitochondrial autophagy [184]. Apigenin attenuated copper-mediated β-amyloid neurotoxicity in an Alzheimer’s disease cell model, through regulation of redox imbalance, preservation of mitochondrial function, MAPK pathway inhibition, and inhibition of neuronal apoptosis [185]. Apigenin may also be beneficial in immune disorders and chronic asthma. To this end, a non-eosinophilic inflammation, dysregulated immune homeostasis, and mitochondria-mediated airway epithelial cell apoptosis were ameliorated via the ROS/ASK1 (apoptosis signal-regulating kinase 1)/MAPK pathway after apigenin treatment [186]. Clinical studies evaluating apigenin are being prepared/ongoing, regarding its effect in rheumatoid arthritis, Parkinson’s disease, and improvement of organ function in elderly patients with sepsis [187–189]. However, it is not likely that effective plasma concentrations may be achieved by dietary ingestion of apigenin-containing plant materials; therefore, strategies to improve the delivery and access of apigenin by the target tissues are needed, such as use of liposomes or nanostructured lipid carriers [190, 191].

### Vitamin D

Vitamin D is a fat-soluble vitamin represented by two main forms, D_2_ (ergocalciferol, (3S,5Z,7E,22E)-9,10-secoergosta-5,7,10(19),22-tetraen-3-ol) and D_3_ (cholecalciferol, (3S,5Z,7E)-9,10-secocholesta-5,7,10(19)-trien-3-ol), which are well-absorbed in the small intestine [192, 193]. Ergocalciferol occurs in plants and yeast; on the other hand, cholecalciferol is present in animal sources. Moreover, vitamin D is available from sun exposure. In the skin, vitamin D is synthesized from 7-dehydrocholesterol by ultraviolet B (UVB) light from the sun [194]. Food sources of vitamin D_3_ include fish and seafood (mainly fatty fish like salmon, mackerel, trout, and herring) and also egg yolk [194].

Vitamin D through the specific receptor (vitamin D receptor, VDR) affects the function of the central nervous system (neurons, microglia, and astrocytes), the cardiovascular system (vascular smooth muscle cells, pericytes, endothelial cells, and cardiomyocytes), tissue healing (fibroblasts), and epithelium [195].

Within the central nervous system (CNS), vitamin D signaling plays a fundamental role in brain development, neuroprotection, neurotransmission, and immunomodulation. In addition, vitamin D represents an essential protective mechanism in the CNS, which enhances mitochondrial brain energy metabolism [196]. Vitamin D–linked molecular pathways in the CNS are triggered through the VDR, a zinc-finger protein structure within the nuclear receptor superfamily. However, the precise signaling pathways by which vitamin D realizes mentioned activities in the CNS are poorly understood [197]. In the cardiovascular system, 1,25(OH)_2_D_3_ vitamin form inhibits renin gene expression by affecting the cyclic AMP signaling, a pathway that shows a crucial activity in renin biosynthesis/release as a response to different physiological stimuli [198]. In this regard, vitamin D–mitochondrial function cross-talk could affect the cellular signaling involved in the development of hypertension [199]. Recent data showed that the beneficial effects of vitamin D in mitochondria-mediated cardiovascular effects are observed mainly in cardiomyocytes [200]. In addition, 1,25(OH)_2_D_3_ reduces fibroblast differentiation and ECM formation via Smad2/3-dependent TGF-β1 signaling pathways [201] and the activation of VDR signals suppressed TGFβ-mediated EMT in a model of renal fibrosis [202]. Fiz et al. [203] found that TGFβ/vitamin D interplay positively modulates mitochondrial activity in human pancreatic cancer cells. It was documented that activated VDR inhibits EMT through the modified epithelial mitochondrial function during the process of fibrosis [204].

In addition to above-mentioned mitochondria-relevant functions of vitamin D, activated VDR rewires cell metabolism towards the biosynthetic pathways. Decreased vitamin D levels reduce mitochondrial activity and ATP production from oxidative phosphorylation and increase oxidative stress and inflammation [205, 206]. Vitamin D–controlled mitochondrial health may also have implications for self-renewal capacity of cells [207].

Vitamin D is an important substance for skeletal muscle and bone support in humans. The regeneration of muscle includes complex mechanisms comprising the restoration of mitochondrial functions [207]. More specifically, vitamin D_3_ therapy increased mitochondrial oxidative phosphorylation in muscle cells after exercise in symptomatic, vitamin D–deficient subjects [208]. Another study revealed that vitamin D deficiency is linked with muscle atrophy, increased oxidative stress, and decreased mitochondrial functions in the multifidus muscle in patients [209]. Regarding bone health, hypovitaminosis D represents a risk factor for decreased bone strength in primary mitochondrial disease in human subjects [210].

### Silibinin

Silibinin (or silybin), a (2R,3R)-3,5,7-trihydroxy-2-[(2R,3R)-3-(4-hydroxy-3-methoxyphenyl)-2-(hydroxymethyl) 2,3-dihydrobenzo[b][1,4]dioxin-6-yl]chroman-4-one, is a flavonolignan, a natural polyphenolic flavonoid. Silibinin is a major bioactive component of *Silybum marianum* L. fruit extract called silymarin. Silibinin itself is a mixture of two diastereomers, silybin A and silybin *B*, in approximately equimolar ratio. Silibinin is not only the major silymarin compound but is also the most bioactive substance of this extract which is widely used as a traditional medicine for liver diseases in Asia and Europe [211, 212].

Silibinin has been described as a substance with significant pharmacological activities, i.e., hepatoprotective, cardioprotective, neuroprotective, hypocholesterolemic, antioxidant, anticancer, or antiviral [213–219]. In this regard, silibinin has been found to be a potentially effective substance in the modulation of mitochondrial dysfunction and the consequence formation of reactive oxygen/nitrogen species that are included in the initiation and progression of chronic liver disease, age-related neurodegenerative disorders, and other chronic diseases [220, 221].

More specifically, it has been described that the modulation of AMPK signaling of mitochondrial function may be a key in the development of human steatohepatitis. Salomone et al. reported that silibinin renews cellular NAD^+^ levels and activates the SIRT1/AMPKp pathway in non-alcoholic fatty liver [222]. In another study, silibinin suppressed ischemia–reperfusion injury–induced mitochondrial dysfunction and ER stress. This silibinin-induced cardioprotection was linked with downregulated apoptosis, inflammation, and oxidative stress via the inhibition of NF-κB signaling [223]. In addition, neuroprotective activities of silibinin were found after cerebral hypoxia/reoxygenation injury through the activation of the GAS6/Axl cell signaling [215]. The JNK/SAPK-activated signaling after silibinin treatment was responsible for mitochondrial apoptosis in pancreatic carcinoma [224]. Finally, silibinin suppressed the presence of endometriotic lesions through the downregulation of pro-inflammatory cytokines in mice via the anti-inflammatory modulation of MAPK signaling [225].

The above-mentioned findings point to silibinin as a clinically promising substance that can be further developed as a therapeutic candidate for the prevention and treatment of chronic diseases in humans. The characteristic of silibinin to alleviate mitochondrial failure is part of its beneficial properties; however, future clinical research with larger patient samples and longer duration is needed to elucidate its clinical contribution in this regard [226].

### Allicin

Allicin, 2-propene-1-sulfinothioic acid S-2-propenyl ester, is an organosulfur compound present in garlic. It is produced when the garlic compound alliin is enzymatically changed by alliinase [227].

In clinical sphere, allicin has been assessed in several human diagnoses, such as the management of cold, hypertension, rheumatoid arthritis, hypercholesterolemia, diabetes, and nephrotoxicity, or its protective role was evaluated in atherosclerosis and cancer disease. Numerous research data described possible cardioprotective effects by causing vasorelaxation and the moderation of certain pathological conditions of cardiovascular diseases, including cardiac hypertrophy, platelet aggregation, angiogenesis, and metabolic disorders such as hyperlipidemia or hyperglycemia [228, 229].

Allicin was documented as a mitochondrial dysfunction–alleviating substance in different cell types [230–232]. In this regard, allicin with its antibacterial activities ameliorated sepsis-triggered acute kidney injury via activation of Nrf2/HO-1 cell signaling [233]. Using an in vitro model of primary porcine cardiomyocytes, allicin decreased mitochondrial injury, myocardial apoptosis, and inflammation during hypoxia-reoxygenation via modulation of several signaling pathways including PPARγ coactivator 1α, endothelial NO synthase, endothelin-1, HIF-1α, and TGF [232]. In the model of intervertebral disc degeneration, allicin protected the nucleus pulposus cells against advanced oxidation protein product–modulated mitochondrial dysfunction and oxidative stress through the downregulation of the p38-MAPK signaling pathway [234]. Regarding nephroprotective effects, allicin improved mitochondrial dysfunction in acrylamide-induced nephrotoxicity in rats through the upregulation of the SIRT1 signaling pathway [235]. In another study evaluating the cardioprotective effects of allicin, the suppression of lipopolysaccharide-induced oxidative stress and inflammation was observed in human umbilical vein endothelial cells. The authors summarized that these effects were caused by the alleviation of mitochondrial dysfunction and increased activity of Nrf2 signaling [236]. Besides that, allicin shows sensitizing activity on hepatocellular cancer cells to 5-fluorouracil therapy via the activation of ROS-mediated mitochondrial pathway associated with the modulation of caspase-3/PARP/Bcl-2 signaling [237]. Finally, allicin was responsible for the neuroprotection of PC12 cells against 6-OHDA-triggered mitochondrial dysfunction and oxidative stress through the modulation of mitochondrial dynamics (Opa-1/Fis-1/Drp-1 signaling) [230].

As we mentioned above, allicin shows significant antimicrobial, cardioprotective, neuroprotective, anti-inflammatory, metabolic, or anticancer effects. Based on these data, allicin possesses a clinical potential in developing effective therapeutics. However, due to the absence of valid clinical data so far, it is necessary to define the safe and effective doses of allicin for its application against specific diseases within in-depth evaluations. Only clinical trials and quality control studies can solve the beneficial introduction of allicin into clinical practice [238].

### Ginsenosides

Research has further established that mitochondria are one of the most important targets of ginsenosides.

Ginsenosides are the main active ingredients of medicinal herbs from *Panax* species, such as *Panax ginseng* (Asian, Chinese, or Korean ginseng) and *Panax quinquefolius* (American or North American ginseng). They are found in all the tissues of ginseng, including roots, leaves, stems, or fruit [239]. Based on a chemical structure, ginsenosides can be subdivided into three groups, i.e., protopanaxadiols (PPD-type), protopanaxatriols (PPT-type), and oleanolic acid (OA). With exception of OA, ginsenosides are tetracyclic dammarane-type saponins [240]. The major ginsenosides found in both ginsengs are the Rb1, Rb2, Rb3, Rc, Rd, and Rg3 from PPD type; further, the Rf, Re, Rg1, and Rg2 from PPT-type; and several others [241, 242]. Chemoprotective and chemotherapeutic properties of ginseng saponins have been demonstrated in cancer, diabetes mellitus, cardiovascular, neurodegenerative, or liver diseases [243–245]. In addition, they can support hemostasis of the immune system [246–249].

From a pharmacological point of view, ginsenosides have shown significant antitumor, antioxidant, anti-inflammatory, antihypertensive, antiapoptotic, immunomodulatory, and neuro- and mitochondria-protective effects in a wide range of experimental in vitro and in vivo studies. Their anticancer activity consisted in the induction of apoptosis and inhibition of angiogenesis and metastasis, further in their antiproliferative, anti-inflammatory, and antioxidant effects [246, 250]. Ginsenosides were able modulate various cancer-related pathways, including the p53, NF-κB, MAPK, PI3K/Akt, and ERK1/2 signaling pathways [250–252]. In addition, they affected the expression of several cancer-related miRNAs (miR-18a, miR-128, miR-21, miR-4425, miR-3614-3p, miR-520 h, and others) and thereby significantly suppressed the process of carcinogenesis [244, 253–257]. Anti-inflammatory activity of ginsenosides was shown by inhibition of several pathways, such as the p38/JNK/TBK1, AKT/NF-κB, or p38 MAPK signaling pathways [258–261]. Ginsenosides are also potential modulators of the Akt/CREB/BDNF signaling pathway towards neuroprotective effects [262].

Ginsenosides showed cardio- and neuroprotective effects by regulating mitochondrial energy metabolism, biosynthesis, apoptosis, mitophagy, or the status of membrane channels [245, 263–265]. Many experimental studies also demonstrated that ginsenosides possess significant antioxidant effects and played the important role in maintaining the structural and functional integrity of mitochondria. They were involved in regulating multiple oxidative stress–related signaling pathways, such as the Keap1/Nrf2/ARE, PI3K/Akt, Wnt/β-catenin, and NF-κB signaling pathways [248]. In addition, ginsenoside Rg1 exerted antiapoptotic effect on non-alcoholic fatty liver cells. It downregulated the expression of SGPL1 and Bax proteins and upregulated the expression of Bcl-2, indicating that ginsenosides could promote the stability and integrity of the mitochondrial membrane [266].

Although ginseng is used in clinical settings worldwide, most studies on the pharmacological effects of ginseng and its ginsenosides are at the experimental level. Related evaluation of their clinical application is still rare [267]. Jung et al. [268] have proven a favorable effect of ginseng on mitochondrial function and anabolic hormones in men with metabolic syndrome in their clinical study. The antioxidant effects of ginseng were demonstrated in clinical study of Yang et al. [269] when ginseng significantly decreased the level of serum ROS and methane dicarboxylic aldehyde activity in healthy volunteers. Kwon et al. [270] reported a beneficial effect of ginsenosides on cholesterol homeostasis in postmenopausal women with hypercholesterolemia, suggesting possible chemoprotective effects of ginsenosides against more severe cardiovascular diseases in this group of women. The meta-analysis of randomized clinical trials has showed that ginsenoside Rg3 can enhance drug efficacy and reduce drug-induced toxicity from chemotherapy in advanced non-small cell lung cancer patients [271]. However, further research is also needed to elucidate the underlying mechanism of action, pharmacokinetics, and pharmacodynamics of ginsenosides, to expand theoretical and experimental basis for their clinical interventions.

### Oleanolic acid and its derivatives

Oleanolic acid (OA; (3β)-3-hydroxyolean-12-en-28-oic acid) is a pentacyclic triterpenoid compound of plant origin, commonly found in various types of fruits (especially in olives, apple, grape, loquat, elderberry, jujube), vegetables (garlic, legumes), medicinal plants (*Lantana camara*, *Ligustrum lucidum*, *Panax* sp.), and spices (thyme, clove). From these plant sources, it is most often obtained as a free acid, or an aglycone of triterpenoid saponins [272, 273]. The extensive pharmacological activity of OA has been demonstrated in experimental studies of various chronic diseases, including cancer, cardiovascular diseases, obesity, and diabetes mellitus [272, 274]. However, chemical structure of natural OA significantly reduces its oral bioavailability and limits its direct clinical use in humans [275, 276]. Current research is therefore primarily focused on the preparation of new OA dosage forms (nanoparticles, liposomes, solid dispersions, phospholipid complexes), or the development of its new (semi)synthetic derivatives with better pharmacological properties in terms of potency, toxicity, bioavailability, and solubility [272, 273, 275, 277]. To the best-known derivatives of OA (OADs) belong 2-cyano-3,12-dioxooleana-1,9(11)-dien-28-oic acid (CDDO), CDDO-methyl amide (CDDO-MA), CDDO-methyl ester (CDDO-Me), and CDDO-imidazole (CDDO-Im) as well as vinyl boronates, esters, oximes, and oxadiazole derivatives of OA.

OA and its derivatives (alone or in combination with other pharmaceuticals) have demonstrated significant multi-factorial activity (antitumor, antioxidant, anti-inflammatory, hepatoprotective, hypolipidemic, antihypertensive, antidiabetic, antimicrobial, antiparasitic) and the ability to interfere with multiple signaling pathways [272, 278]. In human cancer cell studies or in vivo models (hepatocellular, breast, lung, and others), OA and its derivatives significantly inhibited the carcinogenesis, reduced the growth and proliferation of cancer cells, induced their apoptosis, showed cytotoxic effects, or inhibited angiogenesis, invasion, and metastasis [279–292]. Within cancer-related signaling, they significantly inhibited the PI3K/Akt/mTOR/NF-κB [292–296], ERK/JNK/p38 MAPK [291, 297], STAT3, and Hedgehog pathways [286]. They also affected the p53 pathway by upregulation of p53 protein expression, p53-mediated cell cycle arrest [280], and p53-dependent apoptosis [298]. In other studies, OA and its derivatives downregulated the Akt/mTOR/S6K and ERK1/2 pathways [282], influenced the miR-122/Cyclin G1/MEF2P pathway by overexpression of tumor suppressive miR-122 [284], or altered the expression of glycolytic enzymes [285]. In addition, they suppressed multi-drug resistance [299, 300], increased the radiosensitivity of tumor cells [301–303], inhibited inflammatory processes [304] or increased the expression levels of antioxidant enzymes [289], and did so via several mechanisms of actions. Several experimental models have been demonstrated their significant protective effects on acute as well as chronic liver injury [275, 305–307]. Hepatoprotective mechanisms of actions of OA and its derivatives may be related to several signaling pathways including those which affecting expression of NRF2-, metallothionein-related genes, or FXR, such as Akt or ERK pathways [275].

In addition to above-mentioned activities, OA and its derivatives possess important mitochondria-relevant functions. They are for example important inducers of mitochondrial-dependent apoptotic pathway. They can alter mitochondrial membrane potential, release caspase activators into the cytoplasm, alter expression levels of pro- as well as antiapoptotic enzymes, and lead to overexpression of ROS and fragmentation of DNA molecule and ultimately to the tumor cells apoptosis [278, 280, 288, 308–310]. Gong et al. [311] also showed the protective effect of OA in in vivo model of cardiac ageing via regulation of mitochondrial integrity.

The most studied OADs in clinical trials is CDDO-Me. Based on experimental studies, it is the important Nrf2 pathway activator and NF-κB pathway inhibitor. In clinical trials, CDDO-Me demonstrated antitumor activity in phase 1 for advanced solid tumors and lymphoma [279]. Its therapeutic effect was also proven in phase 2 and 3 clinical trials for chronic kidney disease through a significant increase of eGFR and reduction of the loss of kidney function [312–314]. In addition, CDDO-Me increased endothelial NO bioavailability and reduces vascular remodeling in a phase 2 trial for connective tissue disease–associated pulmonary arterial hypertension [315]. CDDO-Me has also exhibited positive therapeutic effects in clinical trials for diabetic nephropathy [316].

### Melatonin

Melatonin (N-acetyl-5-methoxytryptamine, N-[2-(5-methoxy-1H-indol-3-yl) ethyl] acetamide) is mostly known as a pineal gland hormone, but in vertebrates, pineal melatonin represents only 5% or less of the total amount generated [317]. Melatonin is an ubiquitous molecule synthesized not only by animal taxa but also by unicellular, bacteria, fungi, and plants [318, 319]. Melatonin content in plants varies greatly (concentrations ranging from 1 ng to 6 μg/g), dietary sources rich in melatonin include coffee beans, goji berries, white radish, apples, cherries, and tomatoes, but even higher levels were found in medicinal and aromatic plants, particularly thyme (38 μg/g), Chinese liquorice, sage, and St. John’s wort [320]. However, phytomelatonin levels depend on ripeness stage and postharvest conservation and, in addition, abiotic stressors modulate its production; therefore, the level may differ within the same plant species depending on environmental conditions [320, 321]. Melatonin intake from animal-source foods is usually lower than from plant food (concentrations ranging in ng/g) [322].

Melatonin regulates numerous physiological processes including circadian rhythms, sleep, metabolism, reproduction, cardiovascular, immune, and hematopoietic systems [323–325]. Melatonin is a well-known ROS/RNS scavenger but exerts its antioxidant activities also through indirect mechanisms, *e.g*., by enhancing the activity of mitochondrial electron transport chain, activation of antioxidative, and inhibition of pro-oxidative enzymes and enhancing the DNA repair [326, 327]. Alleviation of oxidative stress is only one of the many properties of this indoleamine with potential to prevent/reverse various pathological conditions; others include modulation of the cell cycle, apoptosis, modulation of oncogene expression, immunomodulation, and inhibition of angiogenesis [324, 328].

Mitochondria-related beneficial effects of melatonin are well-known [329, 330]. Melatonin protects mitochondria by eliminating ROS, inhibiting the mitochondrial permeability transition pore, and activating uncoupling proteins, thus maintaining the optimal MMP and preserving mitochondrial functions [331]. Melatonin also modulates mitochondrial dynamics which include improvement of mitochondrial biogenesis [332], mitochondrial fission/fusion [333], and autophagy/mitophagy [334]. Melatonin promoted AMPK phosphorylation and accelerated the translocation of PINK1 (PTEN-induced kinase 1) and Parkin to the mitochondria, thereby activating mitophagy [335]. Another study showed that melatonin promotes mitophagy through upregulation of SIRT1 and downregulation of forkhead transcription factors class O (type1) (FoxO1) [336]. Mitophagy maintains functional mitochondria by degradation of disrupted or redundant mitochondria, but, on the other hand, dysregulated mitophagy may result in mitochondrial dysfunction and thus promote chronic disorders, including ischemia/reperfusion-related diseases in vital organs, neurodegenerative conditions, and cancer [337]. Induction or inhibition of autophagy/mitophagy by melatonin is based on cellular needs and oxidative stress levels [338–341].

Human data indicate that melatonin acts as a cardioprotective [342] and neuroprotective molecule in disorders where oxidative damage has been implicated as a common link [343]. However, data regarding the actual impact of melatonin on mitochondrial function from clinical studies are scarce. In patients with Parkinson’s disease, high melatonin dose (25 mg) recovered platelet mitochondrial function and diminished serum markers of oxidative stress [344]. Supplementation with melatonin promoted the development of immature human oocytes retrieved from the controlled ovarian hyperstimulation by protecting mitochondrial function; upregulation of genes associated with ATP generation, increase in the MMP, and decrease in the intracellular ROS and Ca^2+^ levels was found [345]. Assessment of the anticancer efficacy of melatonin in clinical studies was inconsistent due to varied dosages and times of administration [346]. Nevertheless, as cancer and other pathologies such as diabetes, gastrointestinal, neurodegenerative, and other diseases are related to mitochondrial dysfunction, regulation of mitochondrial homeostasis by melatonin may play a significant role in these pathologies [347].

In general population, melatonin is used for jet lag alleviation and sleep induction; over-the-counter supplements usually contain 1–5 mg. Melatonin short-term use is considered safe, even in higher doses; only mild adverse effects, such as dizziness, headache, nausea, and sleepiness have been reported. Safety of long-term use and use in children and adolescents, pregnant and breast-feeding women, and people with specific medication, however, requires further investigation [348–351].

## Coenzyme Q_10_

Coenzyme Q_10_ (CoQ_10_) originally isolated from beef heart is a vitamin-like molecule also synthesized by human cells acts an essential element of the mitochondrial respiratory chain, mobile electron carrier, membrane stabilizer, and redox-regulated modulator of molecular signaling and gene expression mechanisms [352, 353]. Cell membranes and mitochondria both contain reduced (ubiquinol) and oxidized (ubiquinone) forms of CoQ_10_. Fish, chicken legs, herring, and trout are usual foods sources of CoQ_10_: 3–5 mg is estimated to be physiologically consumed daily [354, 355]. In tissues with particularly high energy demands such as the heart, skeletal muscles, and neurons, CoQ_10_ is abundantly concentrated indicating its prioritized bioenergetic function [352, 356]. Further, CoQ_10_’s antioxidant activity is attributed to its reduced form capable to mitigate oxidative damage to sub/cellular structures and lipid peroxidation and to regenerate other antioxidants such as vitamins C and E in the body. CoQ_10_ exhibits anti-inflammatory action by modulating NF-κB-dependent molecular pathway [355].

CoQ_10_ deficiencies may occur due to genetic and mitochondrial diseases, imbalanced oxidative stress and suboptimal dietary habits, accelerated ageing, carcinogenesis, and health adverse effects of statin treatments, among others. Low levels of CoQ_10_ are characteristic for mitochondriopathies, diabetes mellitus, cancers, cardiovascular and cerebrovascular diseases including but not restricted to the heart failure, myocardial infarction, migraine, chronic kidney disease, and hypertension, neurodegenerative disorders including Alzheimer’s and Parkinson’s disease, and muscular dystrophy. In contrast, CoQ_10_ treatment improves muscle endurance, decreased fatigue during daily duties, and reduced serum lactate and pyruvate levels. Treatment with oral CoQ_10_ is often used to support mitochondrial health, sufficient energy, and antioxidant activity to prevent and mitigate many medical conditions [355, 357].

## L-Carnitine

Carnitine (3-hydroxy-4-N-trimethylammoniobutanoate) profiling is a useful diagnostic and prognostic tool for a wide range of diseases involving carnitine deficiency and poor fatty acid oxidation such as patients diagnosed with metallic disorders, e.g., diabetes mellitus predisposed to cascading complications, neurotoxicity, and mental disorders [1]. Carnitine is synthesized in the human body using amino acids: L-lysine and L-methionine [358]. Vitamin C, vitamin B_6_, niacin, and reduced iron act as cofactors in the synthesis of carnitine [359]. Since only 25% of carnitine is synthesized endogenously, humans obtain carnitine substantially from their diet, primarily from meat, poultry, fish, and dairy products [360]. The biologically active stereoisomer, L-carnitine, transports long-chain fatty acids from the cytoplasm to mitochondria for the energy production via beta-oxidation pathway via acetyl-CoA formation, removing short- and medium-chain fatty acids protecting mitochondria against their accumulation. LC controls pyruvate dehydrogenase activity by interacting with acetyl-CoA and maintaining acetyl-CoA/CoA ratios in the cell [359, 361]. LC also regulates the metabolic processes engaged in skeletal muscle protein balance: proteolysis and protein synthesis. Contextually, LC may be able to mitigate muscle damage that results from exercise because of its antioxidant and anti-inflammatory properties [361]. Acetylated LC form, acetyl-L-carnitine (ALC), is a well-tolerated nutraceutical substance. As a well-acknowledged “mitochondrial nutrient,” ALC mitigates mitochondrial stress, therefore, protecting mitochondrial populations against burnout. Consequently, ALC demonstrates beneficial effects as a potent protector against stress-related disorders and ageing. Hence, ALC dietary supplementation is considered a promising approach for targeted treatments of severe medical conditions including heart failure, angina pectoris, obesity with complications, cancers, and diabetes mellitus with comorbidities [362]. Last but not least, physiologic ALC concentrations are essential for high CNS performance considering their brain antiageing effects and direct involvement of antidepressant mechanisms including serotonergic, noradrenergic, and GABA neurotransmission pathways [1].

## Creatine

Creatine plays a central role in all high energy demanding systems such as the CNS and muscular system under intensive sport training; these effects of creatine are per evidence associated with its bioenergetic role in the mitochondria [363]. The entire spectrum of the systemic creatine involvement includes direct and indirect activities as an antioxidant and immuno- and neuromodulatory agent. Basically, human cells store only a small amount of ATP. Through the degradation of phosphocreatine, energy is released which is utilized for resynthesizing ADP and Pi back to ATP to preserve cellular function until glycolysis in the cytosol and oxidative phosphorylation in the mitochondria can generate adequate amounts of ATP for metabolic requirements. As an essential component of cell bioenergetics, creatine also facilitates the movement of Pi from mitochondria into the cytosol to form phosphocreatine (i.e., the creatine phosphate shuttle). As a result, phosphocreatine donates its phosphate to ADP to restore ATP for cellular functions, allowing creatine in the cytosol to diffuse back into the mitochondria for transport of the next phosphate farther from its production site. An increase in muscle phosphocreatine and creatine serves as an energy source to meet anaerobic energy demands, thereby providing a vital energy source especially during ischemia, injury, or impairment of mitochondrial function [364, 365].

Creatine is one of the most extensively used dietary supplements globally, with a market value estimated to reach $520 million by 2024 [366]. After being discovered in 1832 by French chemist Michel Eugène Chevreul as a constituent of skeletal muscle, creatine was studied as a food additive in the early 1990s. Creatine supplementation remains unquestionably crucial for boosting exercise performance. In recent years, novel studies have provided evidence of its health-promoting properties outside of the athletic field, for example, in the treatment of neuromuscular and cardiometabolic diseases, posttraumatic rehabilitation, and ageing [367]. The most common source of creatine is animal-based foods, such as human breast milk, infant formulas, meat, poultry, and fish, as well as dietary supplements that contain synthetically prepared and purified creatine, primarily in the form of creatine monohydrate. In contrast, all plant-based foods do not contain creatine [368]. Result of the recent study in the US population reported possible creatine shortage from food with the average intake almost 50% lower than anticipated. At the same time, about 60% of young individuals consumed creatine below a threshold level of 1 g per day, and 17% consumed zero creatine. Lower meat consumption among the general population may contribute to inadequate creatine consumption [369].

Under various physiological (e.g., exercise, pregnancy, and lactation) and pathological circumstances (e.g., tissue trauma, ischemia, and diabetes), endogenous creatine synthesis is inadequate for humans [370]. Taking creatine supplements first boosts skeletal muscle strength, power, and mass, particularly in athletes and bodybuilders who consume little meat [370]. Another benefit of creatine supplementation in elderly subjects is the amelioration of sarcopenia, a condition marked by loss of muscle mass and function [371]. Further, creatine supplementation improves the health of patients with neurological and muscular disorders [372]. So a sufficient intake of dietary creatine may be vital for the maintenance of homeostasis and optimal health in humans, especially for vegan athletes with insufficient consumption of creatine and its precursors (arginine, methionine, and glycine). Moreover, by storing arginine, glycine, and methionine, creatine supplements enable other important metabolic pathways such as protein synthesis, nitric oxide, and glutathione production [368].

Finally, creatine supplementation reduces mitochondrial oxidative damage induced by exposure to neuro-/toxic substances [373]. By enhancing the activity of antioxidant enzymes, creatine contributes to ROS and RNS elimination that benefits metabolism of amino acids susceptible to free radical oxidation such as arginine, glycine, and methionine [374]. Health beneficial properties of creatine are summarized in Table 1.

**Table 1:** Evidence-based health beneficial properties of creatine [367]

Immunomodulatory effects	• Altering production and/or expression TLR • Influencing cytokine via the NF-κB signaling pathway • Attenuating pulmonary and systemic effects of lung ischemia in reperfusion injury • Improving rehabilitative outcomes in patients with cystic fibrosis and COPD • Reducing of pro-inflammatory cytokines (e.g., IL-6) and other markers of inflammation (e.g., TNF-α, PGE2)
Skin health	Tropical creatine application: • Improving cellular energy availability and protection against oxidative and UV damage • Stimulating collagen synthesis, influencing gene expression • Preventing and treating human skin ageing
Fertility	• Sperm quality and function • Supporting reproductive health
Antidepressive effects	• Increasing brain creatine and PCr levels • Eliminating corticosterone-induced depressive-like behaviors • Improving verbal fluency tests
Anticancer properties	Administration of creatine, methylglyoxal, and ascorbic acid: • Eliminating visible signs of tumor growth • Energy-shuttling function • Improving T cell–based cancer immunotherapies
Muscle mass and body composition	• Increasing muscle mass and strength while decreasing protein degradation and bone resorption markers in older men and postmenopausal women • Improving muscle endurance • Promoting fat mass loss
Cognitive function	• Increasing oxygen utilization and attenuating mental fatigue • Improving cognition and memory
Diabetes	• Improving glucose tolerance to ingesting a standard meal • Increasing GLUT-4 translocation • Enhancing glucose uptake and insulin sensitivity • Reducing in HbA1c levels
Heart disease	• Reducing arrhythmias, ischemia-related damage, and/or heart function in individuals with chronic heart failure • Improving energy availability to heart • Reducing incidence of arrhythmias • Improving myocardial function • Reducing stroke-related damage

*COPD* chronic obstructive pulmonary disease, *GLUT-4* glucose transporter type 4, *HbA1c* glycosylated hemoglobin, *IL* interleukin, *NF-κB* nuclear factor-kappa B, *Pcr* phosphocreatine, *PGE2* prostaglandin E2, *TLR* toll-like receptors, *TNF-α* tumor necrosis factor-α

## Pyrroloquinoline quinone (PQQ)

PQQ is a well-known modulator of the energy-related metabolism and neuroprotector. Corresponding mechanisms reply on cellular signaling pathways and enhanced mitophagy and quality of mitochondrial function [375]. Consequently, PQQ dietary supplementation decreases plasma levels of C-reactive protein and IL-6 as well as urinary methylated amines (e.g., trimethylamine N-oxide) [376] demonstrating also antiobesity effects and protection against obesity-related complications such as inflammation, non-alcoholic fatty liver disease, chronic kidney disease, and type 2 diabetes [377].

PQQ is not biosynthesized by mammals, but trace amounts have been detected in mouse and human tissues at picomolar to nanomolar levels. PQQ is abundant in nature and can be found in a variety of dietary sources, such as fermented soy beans (natto), tea, green peppers, parsley, kiwi fruit, and human milk [378].

PQQ serves as an accessory factor for lactate and possibly other dehydrogenases in oxidation of NADH to nicotinamide adenine dinucleotide (NAD +) [378, 379]. Therefore, the results of PQQ treatment resemble those of cellular NAD + such as maintaining mitochondriogenesis [380].

PQQ is involved in the synthesis of sirtuin-related proteins and by maintaining NAD + levels optimizes NAD + which is a cofactor and cosubstrate of sirtuin [380, 381].

PQQ exposure prolongs the lifespan of models used in the study of ageing, improves cytokine response, and acts as a potent redox cycling agent that aids in the suppression of ROS [382].

It has been demonstrated that PQQ mitigates clinically relevant dysfunctions like ischemia, neurogenic losses, inflammation, and lipotoxicity [382]. It provides neuroprotection and improves memory. In neurological injury, PQQ protects NMDA receptors [383, 384]. It is also reported that PQQ plays an important role in serum lipid metabolism, contributing to heart disease prevention [385]. Furthermore, PQQ’s cardioprotective effects in models of ischemia and reperfusion injury are comparable to those of metoprolol, a β1-selective adrenoceptor antagonist [386]. The use of PQQ reduces jejunal mucosal inflammatory injury in experimental animals by blocking mechanisms related to nuclear factor-kappa B (NF-kB)–related pathways and improving the colonic microbiota disturbance caused by different agents [387]. According to Naveed, inflammation of the gastrointestinal tract is strongly associated with the production of reactive oxygen species. By scavenging reactive oxygen species (ROS), PQQ acts as a protective agent. Furthermore, PQQ reduces the levels of C-reactive protein and interleukin-6 [388].

## L-Carnosine

Carnosine (β-alanylhistidine, β-alanyl-L-histidine) is a dipeptide consisting of two amino acids (β-alanine and L-histidine) known to medical science since the year 1900 [389]. Within the human body, and animal bodies as well, it is mostly contained in the skeletal muscle, brain, and heart [390–392]. Carnosine is essential for the normal functioning of the cell. It restores the normal function of the cell and can extend its life via reducing the damage to telomeres [393, 394]. Carnosine effectively acts against processes that lead to deterioration of the condition or changes in cell structure, thus preventing the occurrence and development of various diseases. Many reactions of reactive oxygen species lead to the deterioration of the vital cell structures, including the ones within the mitochondria; in turn, the carnosine acting as highly effective antioxidant can counteract these damages to a big extent [390, 391, 395]. Carnosine has an inhibitory effect on the process of glycation, thus preventing the occurrence of complications in diabetes—cataracts, atherosclerosis, reduction of kidney function, and other. Further, it prevents carbonylation of proteins leading to gradual disruption of their structure resulting in their complete destruction. Carnosine has the ability to chelate important bivalent metal ions such as copper and zinc [391, 392]. Finally, it contributes to elimination of toxic elements, therewith supporting systemic detoxification of human body and protecting against liver diseases [396].

In summary, L-carnosine-related pathways are linked to mitochondrial function; corresponding mechanisms are involved in protection against ageing; metabolic, cardiovascular, and ocular diseases; and neurodegeneration and involved in curative processes such as wound healing, which, if impaired, is considered a prestage to cascading pathologies [390–392, 395, 397]. To this end, L-carnosine is beneficial for physical activities in gyms and outdoors contributing, therefore, to improved body fitness and high performance of professional athletes. Meat, fish, and eggs are the natural sources of L-carnosine; dietary supplements of L-carnosine are individually recommended depending on corresponding medical (e.g., impaired wound healing) and/or health conditions (e.g., regular body exercise and top athletes).

## Octacosanol

Octacosanol (octacosan-1-ol, CH_3_ (CH_2_)_26_ CH_2_-OH)) is a straight‐chain primary aliphatic saturated alcohol contained, for example, in rice bran, sugarcane, beeswax, wheat, apple, barks, leaves, Antarctic krills, and whole seeds [398, 399].

Octacosanol is a hydrophobic bioactive substance demonstrating several medicinal properties such as antifatigue, antihypoxia, antioxidant, anti-inflammatory, antitumor, and antiageing effects. Further important functions of octacosanol include modulation of the immune system and energy metabolism well-reflected in therapeutic benefits demonstrated for cardiovascular and cerebrovascular diseases, diabetes mellitus, and Parkinson’s disease [399]. It was also found that octacosanol extracted from leaves of Amazonian plant *Sabicea grisea* var. *grisea* has peripheral antinociceptive influence in mice, probably mediated by the alpha 2-adrenergic receptors. Contextually, octacosanol is considered for novel strategies for preventing pain and inflammation [400].

Octacosanol belongs to bioactive compounds effective against skeletal muscle diseases. Octacosanol can help resisting fatigue by modulating muscle-fiber-type transition via muscular energy metabolism. The mechanism is mediated by the Bcl3/TLRs/MAPK signaling pathway [398, 401]. To this end, octacosanol regulates several signaling pathways including AMPK, PI3K/Akt, and MAPK/NF-κB highly relevant for the key physiological functions [399].

It is likely that octacosanol contents are insufficient in usually available diets and its supplementation as the valuable multi-faceted nutraceutical is desirable for health benefits and safe therapeutic approaches tailored to individualized patient profiles [402–404].

## Pterostilbene

Pterostilbene (4-[(*E*)-2-(3,5-dimethoxyphenyl)ethen-1-yl]phenol, trans-3′,5′-dimethoxy-4-stilbenol) is a stilbenoid which is chemically close to the resveratrol but demonstrating an improved bioavailability [405, 406]. The most common natural sources of pterostilbene are blueberries, grape leaves, and vines [405, 407, 408]. Pterostilbene demonstrates health beneficial pharmacological properties including anti-inflammatory, antioxidant, antiapoptotic, neuroprotective, anticancer, antidiabetic, cardioprotective, and antiatherosclerotic ones—all linked to the mitochondrial physiology and protection including mitochondrial biogenesis, homeostasis, and rejuvenation under stress conditions [409]. Pterostilbene is highly protective in suboptimal health conditions demonstrating strong therapeutic properties against ageing, obesity, chronic inflammation, and neurological (analgesics), cardiovascular, metabolic, and hematologic disorders [405, 406]. According to the most recent studies, pterostilbene may be superior to resveratrol in supporting mitochondrial biogenesis and regulating mitochondrial redox homeostasis, thereby preserving mitochondrial function, inhibiting cell apoptosis, and ameliorating oxidative stress–induced intestinal injury [410]. Moreover, pterostilbene is capable to palliate negative health effects caused by mitochondrial mutations and to activate SIRT3 as well as its mitochondrial deacetylase function [411]. Pterostilbene is commercially available as trans-pterostilbene in a dosage which usually corresponds to 50 mg of pterostilbene per capsule.

## Alpha-lipoic acid

α-Lipoic acid (5-(1,2-dithiolan-3-yl)pentanoic acid, ALA, also thioctic acid) is an essential component of mitochondrial structure with a cofactor role in enzymatic reactions associated with aerobic pathways and coordination of energy metabolism [412]. Although ALA is produced by each human cell, this amount is insufficient to cover all the requirements. ALA deficits are covered by diets rich in red meat, liver, heart, kidney, and some vegetables such as spinach broccoli, tomatoes, Brussels sprouts, potatoes, garden peas, and rice bran [413, 414]. ALA dietary supplements are commercially available and in combination with dihydrolipoic acid (DHALA) represent a potent antioxidant system capable of regenerating other antioxidants such as vitamin C, vitamin E, gluthathione, and coenzyme Q10—all essential for mitochondrial physiology and energy metabolism [413, 415].

## Catechins

Catechins are well-known scavengers of ROS and metal ion chelators inhibiting pro-oxidant enzymes while inducing antioxidant and detoxification enzymes and mitigating mitochondrial damage under stress overload. These are the key mechanisms underlying catechins’s protective properties against oxidative stress implicated in accelerated ageing processes and associated pathologies including CVDs, neurodegeneration, cancers, and diabetes with cascading complications [416]. Accumulated research data demonstrate specifically the central role of mitophagy modulation by catechins considered a new therapeutic option [417].

A number of foods and beverages naturally contain catechins, including green and black tea, coffee, berries, grapes, and wine [418]. A number of catechin derivatives found in tea belong to the family of flavonoids called epigallocatechin gallate (EGCG), epicatechin gallate (ECG), epigallocatechin chloride (ECC), and epicatechin chloride (EC) [418]. The catechin content of green tea is much higher than that of black tea, and catechins are particularly abundant in green tea that has not been fermented. The antioxidant capacity of green tea leaves may vary depending on their variety and the place of origin [419]. The health-promoting properties of green tea are mainly due to the presence of various beneficial polyphenols, particularly flavonols and flavanols. Catechin’s antioxidant properties are attributed to its ability to chelate metal ions (specifically copper ions) in redox reactions and neutralize free oxygen radicals [419].

A number of human epidemiological and clinical studies have indicated that polyphenols found in green tea leaves have antitumor properties [420]. Polyphenol’s antitumor properties may be attributed to its suppression of cell division and activation of phase II antioxidant enzymes, such as superoxide dismutase, glutathione-S-transferase, and glutathione peroxidase and reductase [416]. Green tea catechins have been reported to suppress aggressive metastatic cancer including prostate, colon, and lung malignancies [421–423]. Various studies have indicated that polyphenols derived from green tea exhibit antitumor activity by modifying histones, micro-RNA, and DNA methylation [424].

A number of experimental studies have reported protective effects of (green tea) catechins against neurodegenerative processes including Alzheimer’s disease. Corresponding protective mechanisms reply on antioxidative, anti-inflammatory, protein kinase C–related, and neurotransmission-related properties of catechins [425].

Considering catechins’ numerous health-promoting properties, the inclusion of catechin-containing foods and drinks into the daily diet is highly recommended.

## The vitamin B family

This group of vitamins are water-soluble coenzymes essential for a spectrum of metabolic pathways and functions at sub/cellular and organismal levels. To this end, mitochondrial health and functionality are compromized by a deficiency of any member of the B vitamin family [426]. Shortly,

- Vitamin B_1_ (thiamin) is required in the citric acid cycle for the oxidative decarboxylation of the multi-enzyme branched-chain ketoacid dehydrogenase complexes.
- Vitamin B_2_ (riboflavin) is essential for the respiratory chain (flavoenzymes regulation).
- Vitamin B_3_ (niacin) is the natural NAD + booster required for the NADH synthesis to supply protons for oxidative phosphorylation, among others.
- Vitamin B_5_ (pantothenic acid) is needed for the coenzyme A formation, for activity of alpha-ketoglutarate and pyruvate dehydrogenase complexes and ß-oxidation of saturated fatty acids.
- Vitamin B_6_ (pyridoxal) collectively refers to six water-soluble vitamers, in which only pyridoxal 5′-phosphate (PLP) is biologically active form essentially involved in a wide range of metabolic, physiological, and developmental processes of all living organisms.
- Vitamin B_7_ (biotin) acts as the coenzyme of decarboxylases and is essential for gluconeogenesis and ß-oxidation of saturated fatty acids.
- Vitamin B_9_ (folate) enables activation and transfer of one-carbon units for the biosynthesis of purines, thymidine, and methionine; folate-deficiency impairs respiratory chain.
- Vitamin B_12_ (cobalamin) is required for protecting mitochondrial function and neuronal signaling; vitamins B_12_, B_6_, and B_9_ are essential coenzymes of methyltransferase remethylating homocysteine into methionine and methylating biomolecules such as chrDNA and mtDNA (global epigenetic regulation); epigenetic modifications in mtDNA strongly contribute to the development of obesity, diabetes mellitus with cascading pathologies, cancers, CVDs, and neurodegeneration.

### Vitamin B_1_ (thiamine)

The earliest and perhaps best acknowledged interrelationship between nutritional deficits and neurological impairments including cognitive deficits, encephalopathy, and dementia is related to the vitamin B_1_. The pathomechanisms rely on reductions in brain glucose metabolism and compromized mitochondrial health leading to cognitive deficits [427].

Thiamine is only produced by plants, microorganisms, and some fungi. Humans and animals acquire thiamine from nutrition, while intestinal bacteria produce insufficient amounts of it [428].

Dietary thiamine requirements vary based on age, gender, and lifestyle, with an adult requiring 1.1–1.5 mg/day. Plant-derived foods such as enriched bread, whole wheat cereals, peas, beans, nuts, and brown rice contain thiamine (primarily in the form of thiamine monophosphate). It is also found in animal products (mainly in the form of thiamine pyrophosphate) such as pork, beef, and pork loin [428]. Since the 1940s, processed cereal products (e.g., flour, bread, cereals) have been enriched with thiamin. During food processing, thiamin can be lost as a result of high temperatures [426].

Thiamine acts as an essential cofactor in central metabolic pathways such as in the citric acid cycle for the oxidative decarboxylation of the multi-enzyme branched-chain ketoacid dehydrogenase complexes [426]. Further, thiamine exhibits significant antioxidant properties: as a ROS scavenger, thiamine neutralizes hydroxyl radicals (HO•) more effectively than hydroperoxyl radicals (HOO•), therewith protecting cells against a variety of toxic agents [429]. However, under chronic stress overload, the level of thiamine, thiamine phosphates, and thiamine-dependent enzymes may get diminished leading to redox imbalance, e.g., seen in neurodegeneration [430]. Thiamine deficiency reduces the activity of thiamine-dependent enzymes, notably α-ketoglutarate dehydrogenase complex, resulting in mitochondrial dysfunction, which leads to reduced activity of the tricarboxylic acid cycle in endothelial cells, astrocytes, and microglia.

Thiamine deficiency can be develop within 2–3 weeks of inadequate intake. Humans are therefore extremely susceptible to thiamine deficiency [431]. In developed societies, chronic alcohol consumption is the most prevalent cause of acute thiamine deficiency. There are several reasons for this, including (I) poor intake, such as chronic alcoholism, a poor diet, or gastric bypass surgery; (II) insufficient absorption, including gastric bypass surgery, vomiting, neoplastic hyperplasia, malnutrition, and malabsorption syndrome; (III) increased loss, due to diarrhea, vomiting, hemodialysis, diuretic drug use, systemic illnesses, infections, and sepsis; and (IV) decreased utilization, such as pregnancy, lactation, decreased enzyme activity, and hyperthyroidism. Deficit of this nutrient in the human diet leads to impaired glucose metabolism, disruption of bioenergetic processes, mitochondrial dysfunction, lactic acidosis (the result of mitochondrial dysfunction of pyruvate dehydrogenase), suppressed DNA synthesis due to low transketolase activity and ribose-5-phosphate synthesis in the pentose phosphate pathway, and disrupted neurotransmitter synthesis. The most common pathology caused by a deficiency is beriberi, a cardiomyopathy characterized by edema and lactic acidosis, as well as Wernicke-Korsakoff syndrome and Wernicke’s encephalopathy. Thiamine deficiency is involved in pathomechanisms of Alzheimer’s, Parkinson’s, and Huntington’s diseases [426, 428, 432, 433].

### Vitamin B_2_ (riboflavin)

Due to the relevance of riboflavin and thiamine to mitochondrial functionality, both vitamins currently undergo clinical evaluation for treatment of mitochondrial diseases. Corresponding strategies consider the use of agents enhancing electron transfer chain function. The medication cocktail comprises coenzyme Q10, riboflavin, thiamine, idebenone, and dichloroacetate [4].

Vitamin B_2_ was first identified by Blyth in 1879 as a yellow pigment present in milk [434]. It is a water-soluble and heat-stable vitamin degraded by the light exposure. Riboflavin can be found in a broad range of foods and natural sources, including milk, organ meats, eggs, fish, nuts, certain fruits and legumes, wild rice, mushrooms, dark green leafy vegetables, yeast, beer, cheese, and dietary supplements [434, 435]. Due to its limited absorption in humans, riboflavin must be obtained through a balanced diet to prevent riboflavinosis, which can result in symptoms such as cheilitis, sore tongue, and a scaly rash on the scrotum or vulva [436]. Preclinical models demonstrated that supplementation with riboflavin significantly extends the lifespan and reproduction capacity [437]. Additionally, riboflavin stimulates the synthesis of normal extracellular matrix and decreases reactive oxygen species levels in keratoconus [438].

**Fig. 1:**
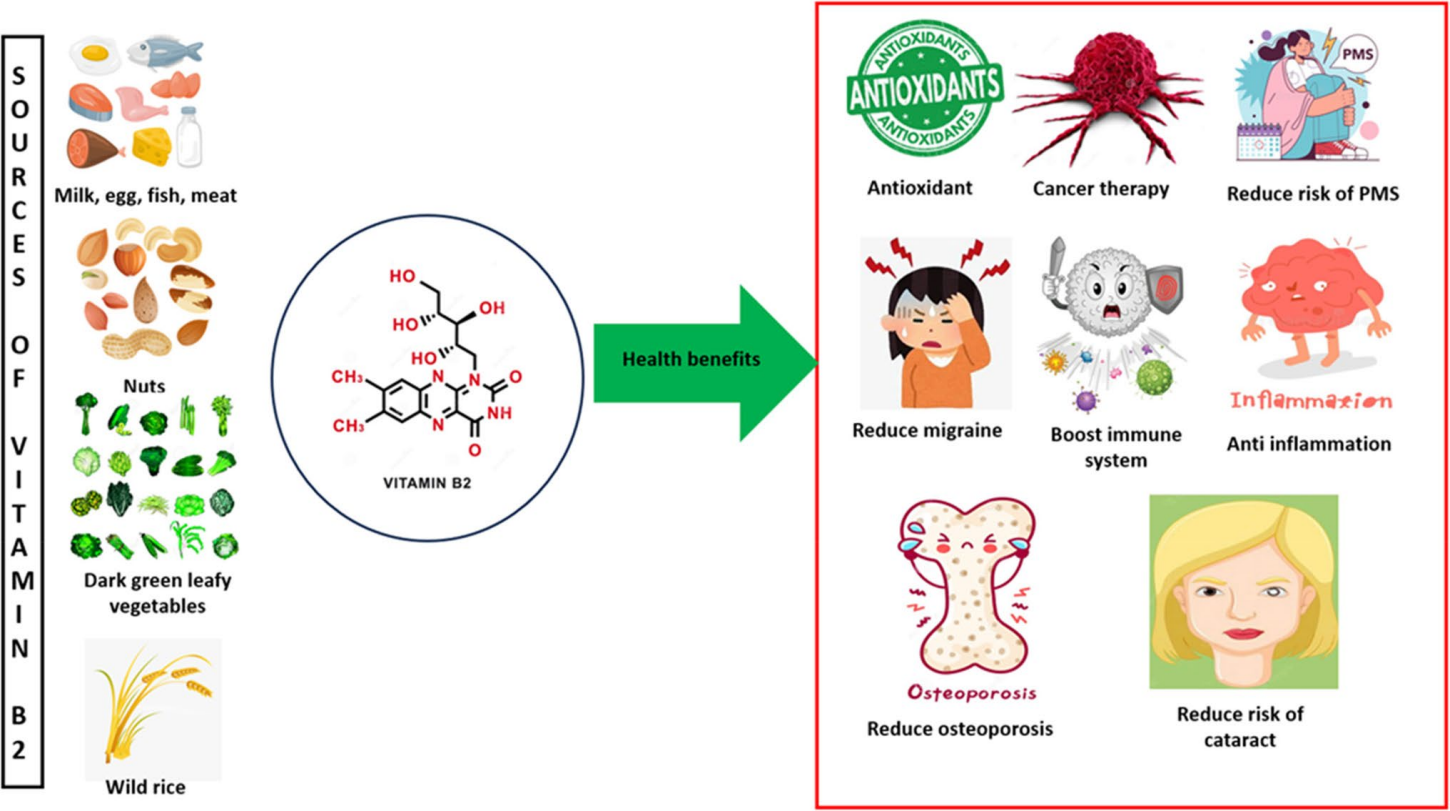
Riboflavin is highly relevant for targeted prevention [435] (Suwannasom et al., 2020)

Both flavin mononucleotide (FMN) and flavin adenine dinucleotide (FAD) function as essential cofactors in numerous enzymatic reactions in all prokaryotic and eukaryotic cells modulating activities of superoxide dismutase, catalase and glutathione peroxidase, and cellular redox status, among others [437, 439]. FMN and FAD play an important role in bioenergetics, photochemistry, bioluminescence, redox homeostasis, chromatin remodeling, DNA repair, protein folding, and other processes [440] highly relevant for preventing a wide range of stress-related medical conditions including chronic inflammation, headaches, anemia, cancer, hyperglycaemia, hypertension, and diabetes, as summarized in Fig. 1. A riboflavin deficiency adversely impacts iron absorption, tryptophan metabolism, mitochondrial function, the gut, brain, and other vitamin metabolism such as vitamins B_6_ and B_12_ [435, 441, 442].

### Vitamin B_3_ (niacin)

Niacin is the natural NAD + booster required for the NADH synthesis to supply protons for oxidative phosphorylation, among others. As soon as nicotinamide enters the cells, it is primarily metabolized into NAD + , an electron carrier that is essential to the formation of ATP through mitochondrial respiration or glycolysis, and NADP + , which acts as a hydrogen donor in fatty acid or steroid synthesis or as a coenzyme in pentose synthesis in the pentose phosphate cycle. There are a variety of foods that contain niacin and nicotinamide, particularly fresh vegetables, milk, meat, whole or enriched grain cereal, and eggs [443]. A large portion of uncooked foods contain NAD + and NADP + , which are hydrolyzed enzymatically to nicotinamide during cooking. Dietary niacin and nicotinamide are taken up from the stomach and intestine through sodium-dependent and passive diffusion. Tryptophan, a component of 1% of protein in the diet, can be turned endogenously into niacin via the kynurenine pathway and quinolinate [444]. This provides most of the body’s niacin demands. To convert tryptophan to niacin, vitamins B_2_ and B_6_ and iron are needed as cofactors. Subsequently, niacin and quinolinate are converted into nicotinic acid ribonucleotides and then into NAD + [443]. Humans with severe nicotinamide deficiency develop pellagra (Italian “pelle agra”; “rough skin”), which is now most prevalent among malnourished alcoholics, but was formerly common in communities dependent upon corn-based diets.

A series of studies have linked altered nicotinamide levels with CNS deficits, Alzheimer’s disease, Parkinson’s disease, and Huntington’s disease; nicotinamide treatment protects against neuronal injury, brain ischemia, and neurodegeneration and demonstrates behavioral recovery in animal models [445].

Further, several micronutrient deficiencies including nicotinamide, riboflavin, zinc, and magnesium have been associated with increased rates of esophageal cancer in China and Italy [446, 447]. There is also an increased risk of oral, gastric, and colon cancer associated with low dietary niacin intake, along with esophageal dysplasia. Based on analysis of a large Western population in the Malmo Diet and Cancer Study in Sweden, approximately 15–20% were deficient in niacin [448]. Western populations are uncommonly affected by severe niacin deficiency that results in pellagra, but individuals at risk of pellagra may have suboptimal niacin intake, such as cancer patients and those exposed to genotoxic agents such as ionizing radiation, ultraviolet radiation, and alkylating agents [444]. NAD + requirements are higher in tissues with high cellular turnover, such as breast, lung, and skin. As a result, they likely require higher doses of NAD + precursors when exposed to genotoxicity [449].

### Vitamin B_6_ (pyridoxal 5′-phosphate)

The fundamental role of the pyridoxal 5′-phosphate enzyme cofactor in human metabolism is well-established through more than 70 years of intensive research history in the field. Vitamin B_6_ deficiency is associated with compromized mitochondrial health, CVDs (caused by impaired homocysteine metabolism and biosynthesis of vasomodulatory polyamines), epilepsy, and neuromuscular and neurological disorders [450]. To this end, neurological disorders and visceral damage are functionally interrelated. The so-called polyglutamine (polyQ) toxicity leading to the neuronal loss and extensive injury of non-neuronal cells is mediated through mitochondria damage and includes the crucial role of the gut-brain axis and gut microbes in development and progression of neurological disorders. To this end, preclinical studies demonstrated mitochondrial protection by vitamin B_6_ supplementation in fat bodies, indicating a promising preventive and therapeutic strategy for maintaining mitochondrial health and effective treatment of the polyQ-induced cellular toxicity [451].

### Vitamin B_9_ (folate)

Folate-based pathways enable activating and transferring one-carbon units for the biosynthesis of purines, thymidine, and methionine. Antifolates are important immunosuppressive and anticancer agents. Mitochondrial folate–associated enzymes are highly upregulated in quickly proliferating cancer cells that reflects the needs of mitochondria in folate-bound one-carbon units for a range of metabolic processes [452]. For example, the loss of the catalytic activity of the mitochondrial folate enzyme serine hydroxymethyltransferase 2 (SHMT2) leads to impaired mitochondrial translation–dependent on tRNA methylation and defective oxidative phosphorylation in human cells. Contextually, targeted antifolate treatments impair the respiratory chain and ATP production. The mechanism of an altered epigenetic control due to abnormal chromosomal and mitochondrial DNA methylation linked to the folate deficiency and hyperhomocysteinemia (Hhcy) is characteristic for several severe pathologies such as the leading cause of blindness in humans by proliferative diabetic retinopathy demonstrating a vicious circle in uncontrolled ROS production and irreversible damage to chromosomal and mitochondrial DNA [453]. Contextually, the folate-based vitamin therapy targeting Hhcy is a promising 3PM approach in secondary DM care preventing associated pathologies [454, 455].

### Vitamin B_12_ (cobalamin)

Mitochondria are the key players in synaptic neurotransmitter signaling providing ATP, mediating synthesis of bioactive molecules, buffering intracellular calcium, and modulating apoptotic and resilience pathways. To this end, cobalamin is considered an essential pillar among nutrients protecting mitochondrial function and neurotransmitter signaling in the neuronal circuits associated with cognitive and affective behaviors. The essential nutrients’ set includes vitamin B_9_ and B_12_, magnesium, ω3 fatty acids, and antioxidants (vitamin C and zinc) which collectively enhance neurocognitive function demonstrating therapeutic benefits in vulnerable groups predisposed to mood disorders and suicidal behavioral patterns [456].

Vitamin B_12_, a water-soluble vitamin, plays an important role in several biological processes including red blood cell production, DNA synthesis, nervous system function, and maintaining the structural integrity of chromosomes [457]. In addition, as a cofactor, it aids in the synthesis of hormones, proteins, and lipids [458]. Vitamin B_12_ is present in several forms, including cyano-, methyl-, deoxyadenosyl-, and hydroxy-cobalamin. The cyano form, commonly used in supplements, is present in small amounts in food. The other forms of cobalamin can be transformed into the methyl- or 5-deoxyadenosyl forms that are necessary as cofactors for methionine synthase and L-methyl-malonyl-CoA mutase [459].

Vitamin B_12_ is synthesized by specific bacteria in the gastrointestinal tract of animals and is then absorbed by the animal and incorporated into its tissues. Omnivores and carnivores, including humans, obtain B_12_ from animal products like milk, cheese, and eggs. A rich source of cobalamin is the liver, followed by the kidney and heart. Neither natural nor synthetic forms of vitamin B_12_ exist in plant sources. Seaweed and mushrooms are said to contain vitamin B_12_ analogues that are inactive in humans [457, 458]. In the body, cobalamin binds to a protein called transcobalamin II and enters tissue through its receptors. Cobalamin contributes to two enzymatic reactions at the cellular level, methionine synthase and methyl-malonyl-co A mutase [458, 459].

The vitamin B_12_ plays a vital role in DNA synthesis and assists in maintaining the structural stability of key regions of chromosomes, such as centromeres and subtelomeric DNA. As a methyl donor, it is involved in the monocarbonic acid metabolic pathway and contributes to DNA methylation, which is particularly critical during embryogenesis and carcinogenesis. DNA methylation is catalyzed by DNA methyltransferases which transfer methyl groups from S-adenosylmethionine to cytosine. In addition to vitamin B_12_, other methyl group donors like pyridoxal 5′-phosphates and folate are needed as coenzymes for methyltransferase that remethylates homocysteine into methionine which is necessary for the methylation of several biological molecules, including DNA. So, B_12_ can influence epigenetic mechanisms through its role in DNA methylation [460, 461].

**Fig. 2:**
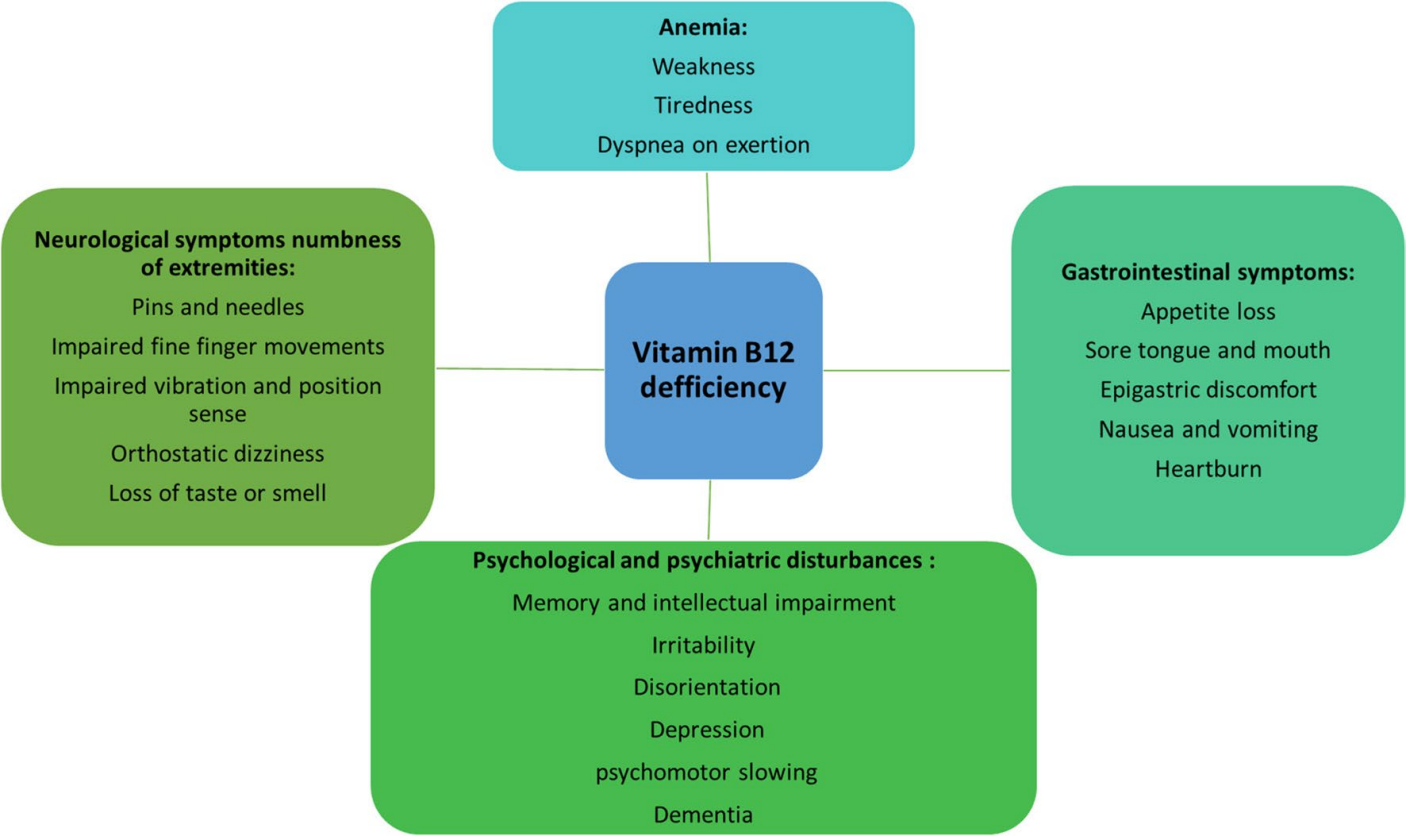
Classical symptoms of vitamin B_12_ deficiency [458]

The main cause of vitamin B_12_ deficiency is avoiding foods from animal origin, which are the only sources of this vitamin [460]. Developing countries, the elderly, and vegetarians are at high risk of subclinical vitamin B_12_ deficiencies. Blood levels of vitamin B_12_ are not the definitive indicator of deficiency, since some people with a deficiency can display normal levels of vitamin B_12_ [462]. More health relevant way to measure vitamin B_12_ activity is to determine blood levels of homocysteine and methylmalonic acid, which are elevated with vitamin B_12_ deficiency. Clinical symptoms of vitamin B_12_ deficiency are typically as an outcome of long-term and chronic malabsorption. The typical features of vitamin B_12_ deficiency are illustrated in Fig. 2 [458]. Medical conditions such as cardiovascular disease, cancers, compromized mental health, and adverse birth outcomes can also be caused by a B_12_ deficiency [462, 463].

### Vitamins B_6_, B_9_, and B_12_

Collectively, vitamins B_6_, B_9_, and B_12_ are essential coenzymes of methyltransferase remethylating homocysteine into methionine and methylating biomolecules such as chrDNA and mtDNA (global epigenetic regulation); epigenetic modifications in mtDNA strongly contribute to the development of obesity, diabetes mellitus with cascading pathologies, cancers, CVDs, and neurodegeneration. To this end, altered methylation and hydroxymethylation patterns in mtDNA have been found in a series of preclinical and clinical studies considering tissues received from patients diagnosed with above-listed pathology [464]. Environmental factors strongly influence mtDNA genome hydro/methylation level leading to compromized mitochondrial health, which can be effectively protected by vitamin supplementation algorithms tailored to individualized patient profiles.

## Trehalose

In in vitro and in vivo analytical sets, trehalose administration was demonstrated to protect cells against cytotoxic effects and injury by activating autophagy and alleviating mitochondrial dysfunction [465, 466]. Cardiovascular and neuroprotective properties of trehalose based on restoring mitophagy and turnover of damaged mitochondria are considered for creating novel therapeutics [467, 468]. Trehalose is a naturally occurring disaccharide in a wide array of organisms including plants, bacteria, yeast, and fungi [469]. A growing number of applications for trehalose are based on its bioprotective properties well-considered in the food, cosmetic, and pharmaceutical industries [470, 471]. Its potential therapeutic application is suggested to mitigate severity of neurodegenerative and cardiometabolic diseases [472] to suppress bone resorption and inflammation and to induce autophagy in relevant medical conditions [471]. Specifically in diabetic patients:

- Modulating the glucose signaling pathway to enhance insulin sensitivity
- Modulating postprandial glucose levels to regulate glucose metabolism
- Regulation of lipid metabolism by controlling postprandial insulin secretion
- Improving activity of pancreatic islet by enhancing pancreatic beta cell function and inhibiting apoptotic processes involved in beta cell malfunction
- Reducing free radical overload and improving insulin resistance by lowering oxidative stress
- Inhibition of inflammatory responses through alleviation of inflammatory mediators

By therapeutic application of trehalose may mitigate secondary complications [473].

## Natural sets of nutraceuticals with multi-faceted beneficial effects

Accumulated data about health protective effects of nutraceuticals led to development of sets applicable to several medical conditions and consideration of natural sources rich in specific nutraceuticals as highlighted below.

### DMG-gold as generalized promotor of metabolic and physical wellness

DMG-gold enhances mitochondrial energy metabolism and acts as an effective protector against stress overload and chronic inflammation. DMG-gold is composed of dimethylglycine (DMG), trimethylglycine (TMG), and vitamins B_1_, B_2_, B_3_, B_6_, and B_12_ [426]. Dimethylglycine boosts the body’s antioxidant capacity by supplying glycine for glutathione synthesis [474, 475]. Furthermore, dimethylglycine, acting as a methyl donor, scavenges excessive free radicals and prevents oxidative stress [476]. The antioxidant properties of B vitamins have also been proven to prevent adverse health effects of oxidative stress overload. According to the accumulated research data, administration of DMG-gold increases detoxifying enzyme activities (GST, NQO1) and glutathione levels, whereas decreasing oxidative stress and inflammation and improving mitochondrial homeostasis. Consequently, reduced serum levels of IL-6 and hepatic inflammatory parameters have been reported [477].

### Ginkgo biloba

**Fig. 3:**
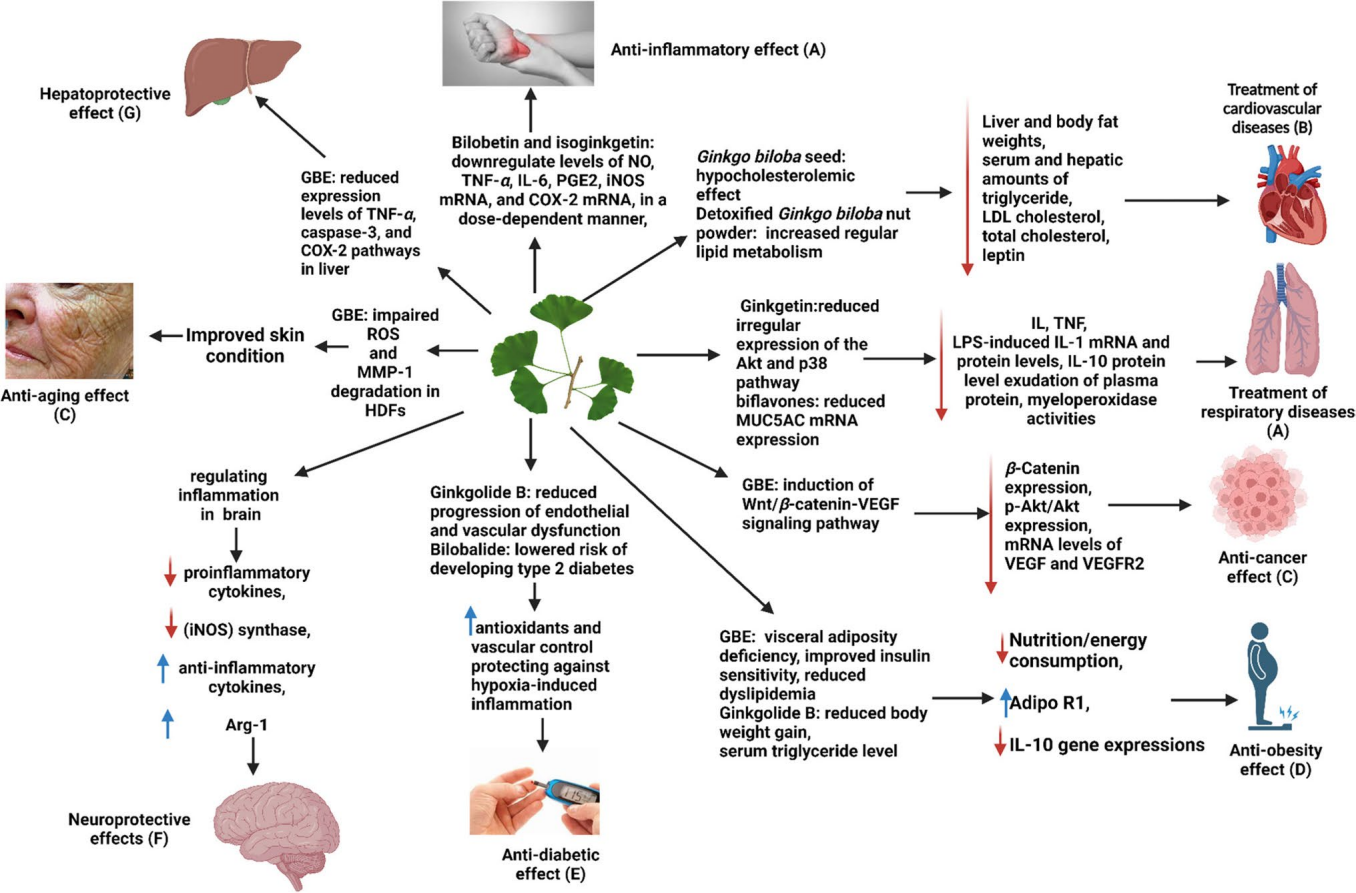
Medicinal properties of *Ginkgo biloba*. Arg-1, arginase-1; COX-2, cyclooxygenase-2; HDF-s, human dermal fibroblasts; IL-6, interleukin-6; iNOS, inducible nitric oxide synthase; LDL, low-density lipoprotein; LPS, lipopolysaccharide; MMP-1, matrix metalloproteinase-1; p-Akt/Akt, protein kinase B (PKB); PGE2, prostaglandin E2; TNF-α, tumor necrosis factor-α; ROS, reactive oxygen species; VEGF, vascular endothelial growth factor; VEGFR2, vascular endothelial growth factor receptor2. **A** [485]; **B** [486]; **C** [479]; **D** [487];** E** [488]; **F** [489]; **G** [490]

Per evidence, *Ginkgo biloba* extract (GBE) activates mitochondrial enzymes that assemble the electron transport system thereby improving mitochondrial respiration, increases production of ATP in neurons, protects brain against ageing, protects mtDNA against damage, and regulates membrane potentials and intramitochondrial Ca^2+^ homeostasis [478, 479]. Although the GBE, specifically the flavonoids bilobalide and ginkgolides B and J, seem to possess the highest protection, it is difficult to discriminate between compounds with the most beneficial properties. Therefore, EGb761, a standardized leaf extract of *Ginkgo biloba*, is usually applied for treatments, when multi-faceted therapeutic effects are considered including scavenger, antioxidant, antibacterial, anti-inflammatory, antiallergic, and anticancer effects [479–482]. GBE is among the most frequently sold medicinal products in US health food stores [483] and used by traditional Chinese medicine for preventing neurodegenerative diseases, fever, coughs, and sputum production, as well as treating skin disorders, gonorrhea, toothaches, among others [484]. Further, EGb761 applied together with grape seed skin extracts, quercetin, green tea, resveratrol, and bilberry extracts synergistically decrease diastolic blood pressure in hypertensive patients [481]. Contextually, GBE protective effects were demonstrated for cardiovascular and retinal systems [479, 482]. Multi-faceted therapeutic effects of GBE are summarized in Fig. 3.

### Propolis

Considering its immunomodulating, anti-inflammatory, antioxidant, anticancer, antibacterial, and antiviral properties, propolis has been proven to be effective in treating various diseases in primary and secondary care as summarized in Table 1. The key mechanisms rely on modulating mitochondrial bioactivities [491–494].

Due to its multi-faceted health beneficial effects, propolis is subjected to a numerous of studies in the pharmaceuticals and nutraceutical areas [495, 496]. Major constituents of propolis are flavonoids; phenolic compounds; polyphenols; terpenes; terpenoids; coumarins; steroids; amino acids; chalcones; essential oils; vitamin complexes A, B, C, and E; and minerals, including aluminum, sodium, potassium, calcium, copper, magnesium, iron, and zinc [495, 497]. Health beneficial properties of propolis are summarized in Table 2.

**Table 2:** Effect of propolis on different medical disorders

Disorders	Measured outcomes	References
Types 2 diabetes	• Decreasing blood glucose, serum insulin levels, and serum HbA1c level • Scavenging free radicals • Modulating blood lipid metabolism • Suppressing intestinal α-glucosidase activity in CHO digestion • Modulating β-cells in islets of Langerhans in the pancreas	[498, 499]
Rheumatoid arthritis	• Suppressing inflammatory cascades by blocking the NF-κB pathway • Reducing ROS by enhancing antioxidants • Regulating functions of immune cells and decreasing cytokines mediated by immune response in T-cells and NF-kB activation • Suppressing DNA synthesis and inflammatory development in T-cells while increasing TGF-β1 in cells • Decreasing mRNA levels of TNF-α	[500–503]
Cardiovascular disease	• Reducing activity of cyclooxygenase, ROS, and NO • Preventing progression of pathological cardiac hypertrophy and heart conditions • Decreasing expression of CD68, TLR4, MMP-9, and TNF-α in the carotid arteries	[504, 505]
Gastrointestinal disorder	• Improving symptoms experienced by IBS patients through reducing visceral motor response • Inhibiting transcription of iNOS gene, influenced by NF-κB • Reducing colon damage, suppressing colonic inflammation, and increasing levels of mucosal and mucin secretion • Improving intestinal barriers, preventing dislocations of bacterial and toxins from the gut to the bloodstream	[506, 507]
Cancer	• Activating caspase cascade mechanisms and releases cytochrome c from mitochondria into cytosol • Preventing cell proliferation • Triggering apoptosis preventing metastases • Inhibiting angiogenesis of cancer cells	[361, 508]
Chronic kidney disease	• Reducing proteinuria in diabetic and non-diabetic CKD patients • Reducing oxidative stress and renal inflammation by inhibiting the TNF-α pathway • Restoring renal function	[509, 510]
Neurological disorders	• Decreasing expression of inflammatory and oxidative markers: TNF-α, NO • Increasing and maintaining antioxidant parameters, including superoxide dismutase • Inhibiting activation of NF-kB, decreasing lipid peroxidation by inhibiting the cyclooxygenase-2 overproduction • Neuroprotection against apoptosis and oxidation	[511, 512]
Asthma	• Antiallergic, antiasthmatic, anti-inflammatory properties, due to inhibitory effects on activation of basophil and mast cells • Decreasing in frequency and severity of asthma attacks via decreasing inflammatory markers, such as TNF-α, IL-4, IL-5, and IL-6	[513, 514]

*CHO* carbohydrate, *CKD* chronic kidney disease, *HbA1c* glycosylated hemoglobin, *IBS* irritable bowel syndrome, *IL* interleukin, *iNOS* nitric oxide synthases, *MMP-9* matrix metalloproteinase-9, *NF-κB* nuclear factor-kappa B, *NO* nitric oxide, *ROS* reactive oxygen species, *TGF-β* transforming growth factor-β1, *TLR4* Toll-like receptor 4, *TNF-α* tumor necrosis factor-α

### Saffron

An effective neuroprotectant saffron targets mitochondria and prevents mitochondrial dysfunction under clinically relevant stress conditions such as ischemia–reperfusion, demonstrating strong antiapoptotic effects [515, 516].

A perennial herb saffron (*Crocus sativus* L.) belongs to the Iridaceae family, widely cultivated in Iran, India, and the Mediterranean areas [517]. Saffron is primarily composed of carbohydrates (starch, gums, pentosans, reducing sugars, pectin, dextrins, pectin, etc.) (63%), amino acids and proteins (12%), moisture (10%), fats (5%), minerals (5%), crude fiber (5%), and vitamins such as vitamin B_1_ and vitamin B_2_. In addition to these components, saffron contains carotenoids, monoterpenes, anthocyanins, and flavonoids [518]. Crocin, crocetin, picrocrocin, and safranal are the principal active compounds in saffron which exhibit a wide spectrum of biological activities including analgesia, antioxidant activity, cardiovascular protection, and inhibition of cancer, diabetes, inflammation, and depression, among others [518, 519].

### Aloe vera

Accumulated research data suggest *Aloe vera* as a potent modulator of mitochondrial functions. The proposed mechanisms rely on the evident modulation of mitophagy and regulation of the Nrf2-mitochondrial axis by the *Aloe vera* extracts [520, 521]. Corresponding beneficial effects have been demonstrated, for example, for mitigation symptoms and severity of inflammatory bowel disease [521]. Further, strong anticancer properties of the *Aloe vera* extracts have been demonstrated in preclinical studies, e.g., for breast and lung carcinomas. Noteworthy, selective effects have been reported with higher apoptotic levels and diminished ATP concentrations in cancer cells compared to non-cancer cells [522].

## Conclusions, recommendations, and outlook in the framework of 3PM

### Nutraceutical sets tailored to individualized patient profiles in primary care

Application of nutraceuticals is beneficial only if meeting needs at individual level. Therefore, health risk assessment and creation of individualized patient profiles are of pivotal importance followed by adapted nutraceutical sets meeting individual needs. Based on the evidence presented above, here, we provide clinically relevant examples of frequent medical conditions, which per evidence require mitochondria-relevant supportive and protective measures as a holistic approach in primary and secondary care. The choice of nutraceutical sets has to be adapted to individualized patient profiles. In order to make the entire approach sound, the essentiality to apply machine learning (AI tool) for creating comprehensive individualized patient profiles followed by treatment algorithms tailored to the created profiles has to be clearly emphasized.

#### Case 1

- A 39-year-old female with characteristic symptoms and signs of the *Flammer syndrome phenotype* (FSF) (low BMI, low BP, frequently cold extremities, migraine with aura, sporadic arrhythmias, tinnitus, hard-to-heal wounds, shifted circadian rhythm, increased sensitivity towards stress and medication, meticulous personality, regular body exercise, etc.); family history of ischemic stroke and normal-tension glaucoma.
- The functional link between FSF, systemic ischemic lesions, energy deficits, and compromized mitochondrial health is evidence-based and detailed in the peer-reviewed scientific literature [11, 523–528].
- Tear fluid analysis demonstrated a significantly decreased mitophagy level compared to the reference values in the corresponding group of age (the know-how of “3PMedicon GmbH” performing internationally validated tests) [529]; the methodology is described elsewhere [528].
- Self-reported anxiety and chronic fatigue symptoms correlate well with the low mitophagy levels recorded.
- Mitochondria-relevant nutraceuticals considered evidently supportive to stabilize health condition of the patient: CoQ_10_ (increases ECT efficacy, mitigates oxidative stress and fatigue, protects mitochondrial health), vitamins B_1_ and B_2_ (increases ETC efficacy), melatonin (increases ETC efficacy, stabilizes sleep patterns, promotes cardio- and neuroprotection), quercetin (promotes mitophagy), L-arginine (stimulates NO production and vasodilatation), vitamin D_3_ (promotes wound healing), and omega-3 fatty acid.
- Additionally, *Ginkgo biloba* and green tea—both demonstrating multi-faceted mitochondrial and systemic protective effects—may be beneficial considering the above-listed specific medical conditions.

#### Case 2

- A 61-year-old female, overweight, prediabetes type 2; family history of CVDs, chronic inflammatory disorders and cancers—all highly relevant to compromized mitochondrial health.
- Self-reported psychosomatic issues.
- The functional link between overweight, metabolic syndrome, cascading complications on one hand, and, on the other hand, compromized mitochondrial health is evidence-based and detailed in the peer-reviewed scientific literature [1, 2].
- Tear fluid analysis demonstrated a significantly increased mitophagy level compared to the reference values in the corresponding group of age (the know-how of “3PMedicon GmbH” performing internationally validated tests) [529]; the methodology is described elsewhere [528].
- Mitochondria-relevant nutraceuticals are considered supportive to stabilize health condition of the patient: CoQ_10_ (anti-inflammatory effects, increases ECT efficacy, mitigates oxidative stress, protects mitochondrial health under pre/diabetic medical conditions), Ketogenic diet in combination with the L-carnitine supplement (promotes ß-oxidation pathways, protects against cascading complications associated with pre/diabetes), resveratrol (strong anti-inflammatory and anticancer effects), and either curcumin or silibinin (strong anti-inflammatory and anticancer protection).
- Further recommendations: couched body exercise to decrease body weight and to increase mitochondrial mass and ATP production; probiotic intake, because gut microbiota has an essential role in the prevention of and protection against both the metabolic disorder and cancer predisposition [530].

#### Case 3

- A 55-year-old male, sedentary lifestyle, overweight, obstructive sleep apnea (OSA); family history: cardiovascular, malignant, and neurodegenerative disorders—all are highly relevant to compromized mitochondrial health.
- Self-reported stress overload and chronic fatigue.
- The functional link between sedentary lifestyle and overweight with cascading complications on one hand and, on the other hand, compromized mitochondrial health is evidence-based and detailed in the peer-reviewed scientific literature [1, 2]. Per evidence, OSA is associated with elevated homocysteine levels in blood [531]; moreover, the severity of OSA is significantly associated with elevated homocysteine levels in patients with ischaemic stroke [532].
- Tear fluid analysis indicated an extremely decreased mitophagy level with a potential to mitochondrial burnout compared to the reference values in the corresponding group of age (the know-how of “3PMedicon GmbH” performing internationally validated tests) [529]; the methodology is described elsewhere [528] that correlates well with the imbalanced stress overload and symptoms of chronic fatigue reported.
- Mitochondria-relevant nutraceuticals considered evidently supportive to stabilize health condition of the patient: CoQ_10_ (anti-inflammatory effects, increases ECT efficacy, mitigates oxidative stress, protects mitochondrial health), Ketogenic diet in combination with L-carnitine supplement (promotes ß-oxidation pathways, protects against cascading complications related to overweight), quercetin (promotes mitophagy), ginsenosides (protective against cancer, diabetes, CVD and, neurodegeneration; mitochondria-protective effects), and vitamins B_12_, B_6_, and B_9_ (remethylation of homocysteine into methionine, epigenetic control, and protection of mtDNA and chrDNA).
- Additionally, *Ginkgo biloba* and green tea—both demonstrating multi-faceted mitochondrial and systemic protective effects—may be beneficial considering the above-listed specific medical conditions.
- Further recommendations: couched body exercises to decrease body weight and to increase mitochondrial mass and ATP production; probiotic intake, because gut microbiota has an essential role in the prevention of and protection against CVDs and cancer predisposition [530].

### Innovative concepts of prehabilitation and rehabilitation approaches based on application of mitochondria-relevant nutraceuticals—clinically relevant examples for secondary care

In the development and progression of critical illnesses as well as in stabilizing health condition and healing of the affected person, mitochondria play the central multi-faceted role including energy metabolism, cell signaling, and regulation of gene expression and cellular calcium levels as well as activation either repair mechanisms or cell death pathways in case of irreversible damage [533]. To this end, damaged mitochondria generate signals, most notably released mtDNA acting systemically as the danger-associated molecular patterns throughout the body [533]. Therefore, to a great extent, physiologic homeostasis and intact functionality of mitochondrial populations are decisive at the organismal level for individual outcomes of life-threatening illnesses. Keeping these facts in mind, innovative concepts of prehabilitation and rehabilitation approaches based on application of mitochondrial health supportive nutraceuticals are clinically relevant for several medical conditions exemplified below.

#### Coronary artery bypass graft surgery

Coronary artery disease causes systemic ischemia and extensive cell death. Revascularization by coronary artery bypass grafting effectively relieves symptoms and decreases mortality in affected patient cohort. However, mitochondrial populations become extensively damaged by ischemia–reperfusion generating excessive ROS and inflammation coupled with compromized metabolic activity and highly increased oxidative stress throughout the body causing systemic toxicity and irreversible organ damage [534]. Contextually, mitochondria-specific protective measures by individualized sets of nutraceutics applied prior to the surgery (prehabilitation) and after the revascularization (rehabilitation) may have enormous health beneficial effects as supported by accumulated evidence [535–537].

#### Ischemic stroke

Mitochondrial dysfunction is the key contributor to the cerebral ischemia–reperfusion damage, and mitochondrial population is an important drug target for treatments of ischemic stroke. Contextually, technological solutions for the mitochondrial transfer by stem cells and delivery of mitochondria-containing extracellular vesicles for ischemic stroke treatments are extensively under consideration [538, 539]. Further, mitophagy was proposed as a specific therapeutic target to advance treatments of ischemic stroke [540]. Collectively, these findings indicate that the concept of mitochondria-based IS rehabilitation utilizing nutraceuticals targeted to specific mitochondrial functions may have enormous cost-effective benefits to health of affected individuals.

#### Treated cancers

A mounting research data demonstrate that chemotherapy introduces a systemic damage to mitochondrial populations throughout the body of treated cancer patients. For example, treatments usually applied to breast cancer are associated with a range of neurotoxic symptoms including pain, chronic fatigue, and cognitive impairments. Even after the treatment completion, these symptoms remain in a significant subset of survivors. Mitochondrial stress, damage, and impairments coupled with neuroinflammation considered the mechanistic pathways underlying neurotoxic symptoms [541]. Besides chronic fatigue, the health adverse effects are further reflected in muscle weakness that can be well-illustrated with breast and prostate patients treated with doxorubicin [542].

To restore mitochondrial functions and to mitigate DOX-induced fatigue, preclinical studies utilized treadmill exercises in a rat model demonstrating beneficial effects resulting in alleviation of muscle weakness and central fatigue. On the other hand, accumulated research data demonstrate that the ROS production significantly elevated by acute body exercises causes whole-body oxidative stress highly relevant for mitochondrial and multi-organ damage [543]. Indeed, recently performed study demonstrates significantly reduced exercise capacity typical for early-stage breast cancer patients treated with chemotherapy [544]. This finding strongly supports the conclusion that in order to be beneficial, body exercise needs algorithms elaborated individually for cancer-treated patients, accompanied with effective support and monitoring of mitochondrial functions.

Contextually, innovative concepts of prehabilitation (prior to chemotherapeutic and irradiation treatments) and rehabilitation (after the treatment completion) elaborated for cancer patient utilizing mitochondria-supportive nutraceuticals are of great clinical relevance in secondary care. In consensus, the Mediterranean diet (MedDiet) in which dietary patterns are known as promoting energy metabolism was recently demonstrated as feasible to attenuate cancer-related fatigue among patients undergoing chemotherapy and irradiation; the efficacy is particularly remarkable for patients recorded with lower MedDiet scores at baseline [545].

## Data Availability

No datasets were generated or analyzed during the current study.

## Abbreviations

6-OHDA: 6-Hydroxydopamine
ADP: Adenosine diphosphate
AKT: Serine/threonine-protein kinase/protein kinase B
ALA: Alpha-lipoic acid
ALC: Acetyl-L-carnitine
AMPK: AMP-activated protein kinase
AP-1: Activator protein 1
ARE: Antioxidant response element
Arg-1: Arginase-1
ASK1: Apoptosis signal-regulating kinase 1
ATP: Adenosine triphosphate
Axl: Tyrosine-protein kinase receptor
Bax: Bcl-2-like protein 4
Bcl-2: B-cell lymphoma 2
Bcl-3: B-cell lymphoma 3
BDNF: Brain-derived neurotrophic factor
CDDO: 2-Cyano-3,12-dioxooleana-1,9(11)-dien-28-oic acid
CHO: Carbohydrate
chrDNA: Chromosomal DNA
CKD: Chronic kidney disease
CNS: Central nervous system
CoA: Coenzyme A
COPD: Chronic obstructive pulmonary disease
CoQ10: Coenzyme Q10
COX-2: Cyclooxygenase-2
CREB: CAMP response element-binding protein
CVDs: Cardiovascular diseases
CYP1A2: Cytochrome P450 1A2
DHALA: Dihydrolipoic acid
DM: Diabetes mellitus
DMG: Dimethylglycine
DNA: Deoxyribonucleic acid
DOX: Doxorubicin
DRP1: Dynamin-related protein 1
EC: Epicatechin chloride
ECC: Epigallocatechin chloride
ECG: Epicatechin gallate
ECM: Extracellular matrix
EGb761: *Ginkgo biloba* Extract commercial product
EGCG: Epigallocatechin gallate
eGFR: Estimated glomerular filtration rate
EMT: Epithelial-mesenchymal transition
eNOS: Endothelial nitric oxide synthase
ERK: Extracellular signal-regulated kinase
ETC: Electron transport chain
FAD: Flavin adenine dinucleotide
FIS1: Mitochondrial fission 1 protein
FMN: Flavin mononucleotide
FOXOs: Forkhead-box transcription factors
FSF: Flammer syndrome phenotype
FXR: Farnesoid X receptor
GABA: Gamma-aminobutyric acid
GAS6: Growth arrest-specific protein 6
GBE: *Ginkgo biloba* Extract
GLUT-4: Glucose transporter type 4
GSK-3β: Glycogen synthase kinase-3 beta
GST: Glutathione S-transferase
HbA1c: Glycosylated hemoglobin
HDFs: Human dermal fibroblasts
Hhcy: Hyperhomocysteinemia
HIF: Hypoxia-inducible factor
HO•: Hydroxyl radicals
HO-1: Heme oxygenase 1
HOO•: Hydroperoxyl radicals
IBS: Irritable bowel syndrome
IKK: Kappa kinase
IL: Interleukin
iNOS: Inducible nitric oxide synthase
JAK: Janus kinase
JNK: C-Jun amino-terminal kinases
Keap1: Kelch-like ECH-associated protein 1
LC: L-Carnitine
LDL: Low-density lipoprotein
LPS: Lipopolysaccharide
MAPK: Mitogen-activated protein kinase
MEF2P: Mitochondrial elongation factor
miRNA: Small non-coding RNA
MMP: Mitochondrial membrane potential
MMP-1: Matrix metalloproteinase-1
MMP-9: Matrix metalloproteinase-9
mRNA: Messenger RNA
mtDNA: Mitochondrial DNA
mTOR: Mammalian target of rapamycin
NAD +: Nicotinamide adenine dinucleotide
NADP +: Nicotinamide adenine dinucleotide phosphate
NADPH: Nicotinamide adenine dinucleotide phosphate
NF-κB: Nuclear factor kappa B
NO: Nitric oxide
Notch1: Neurogenic locus notch homolog protein 1
NQO1: NAD(P)H quinone oxidoreductase 1
NR1C: Nuclear receptor 1C
NRF-1: Nuclear respiratory factor 1
Nrf2: Nuclear factor-erythroid 2-related factor 2
OA: Oleanolic acid
OADs: Oleanolic acid derivatives
OPA1: Mitochondrial dynamin like GTPase
OSA: Obstructive sleep apnea
p38: Mitogen-activated protein kinase family
p53: Protein encoded by *TP53* gene
PARP1: Poly ADP-ribose polymerase 1
PCr: Phosphocreatine
PGC1α: Peroxisome proliferator-activated receptor γ coactivator 1-alpha
PGE2: Prostaglandin E2
PI3K: Phosphoinositide 3-kinase
PINK1: PTEN-induced kinase 1
PKB: Protein kinase B
PLP: Pyridoxal 5′-phosphate
PolyQ: Polyglutamine
PPD: Protopanaxadiol
PPPM/3PM: Predictive preventive personalized medicine
PPT: Protopanaxatriol
PQQ: Pyrroloquinoline quinone
PTEN: Phosphatase and tensin homolog
RAS: Rat sarcoma virus oncogene family
RNA: Ribonucleic acid
RNS: Reactive nitrogen species
ROS: Reactive oxygen species
S6K: Ribosomal protein S6 kinase
SAPK: Stress-activated protein kinases
SGPL1: Sphingosine-1-phosphate lyase 1
SHMT2: Serine hydroxymethyltransferase 2
SIR2: Silent information regulator 2
SIRTs: Sirtuins (NAD^+^-dependent deacetylases)
STAT3: Signal transducer and activator of transcription 3
STK11: Serine/threonine kinase 11
TBK1: TANK-binding kinase 1
TCA: Tricarboxylic acid cycle
TFAM: Mitochondrial transcription factor A
TGF-β: Transforming growth factor-beta
TLR: Toll-like receptor
TMG: Trimethylglycine
TNF-α: Tumor necrosis factor alpha
tRNA: Transfer RNA
UCPs: Uncoupling proteins
UVB: Ultraviolet B light
VDR: Vitamin D receptor
VEGF: Vascular endothelial growth factor
VEGFR2: Vascular endothelial growth factor receptor 2
Wnt: Wingless-related integration site
